# Beta distribution misspecification tests with application to Covid-19 mortality rates in the United States

**DOI:** 10.1371/journal.pone.0274781

**Published:** 2022-09-20

**Authors:** José Jairo Santana-e-Silva, Francisco Cribari-Neto, Klaus L. P. Vasconcellos

**Affiliations:** Departamento de Estatística, Universidade Federal de Pernambuco, Recife, PE, Brazil; University of Sargodha, PAKISTAN

## Abstract

The beta distribution is routinely used to model variables that assume values in the standard unit interval, (0, 1). Several alternative laws have, nonetheless, been proposed in the literature, such as the Kumaraswamy and simplex distributions. A natural and empirically motivated question is: does the beta law provide an adequate representation for a given dataset? We test the null hypothesis that the beta model is correctly specified against the alternative hypothesis that it does not provide an adequate data fit. Our tests are based on the information matrix equality, which only holds when the model is correctly specified. They are thus sensitive to model misspecification. Simulation evidence shows that the tests perform well, especially when coupled with bootstrap resampling. We model state and county Covid-19 mortality rates in the United States. The misspecification tests indicate that the beta law successfully represents Covid-19 death rates when they are computed using either data from prior to the start of the vaccination campaign or data collected when such a campaign was under way. In the latter case, the beta law is only accepted when the negative impact of vaccination reach on death rates is moderate. The beta model is rejected under data heterogeneity, i.e., when mortality rates are computed using information gathered during both time periods.

## Introduction

Several variables of interest assume values in the standard unit interval, (0, 1). This is the case, e.g., of rates, proportions and concentration indices. The beta distribution is commonly used to model such variables. For instance, [1] use the beta law to model the probability of HIV transmission in male-to-female sexual encounters and [2] lists applications of the beta law to engineering. Other applications of the beta distribution can be seen in [3] (gear damage analysis), [4] (relative sunshine duration in Malaysia) and [5] (group-based trajectory modeling of neurological activity of comatose cardiac arrest patients). Additionally, [6] note that “[t]he beta distributions are among the most frequently employed to model theoretical distributions”. It is noted that the beta law arises naturally in ‘normal theory’ since *Z*_1_/(*Z*_1_ + *Z*_2_) is beta distributed if *Z*_1_ and *Z*_2_ are independent chi-squared random variables. The beta distribution can also be obtained as the limiting distribution of eigenvalues ratio in a sequence of random matrices.

Alternative distributions with support in the standard unit interval have been proposed in the literature and have been increasingly used in empirical analyses, such as, e.g., the Kumaraswamy (see [7]) and simplex distributions (see [8]) and more recently, the unit-Weibull (see [9]) and reflected unit Burr XII (see [10]) distributions. It would then be useful to provide practitioners with a test that can be used to determine whether the beta law—which is still the most used model with fractional data—yields an adequate data fit. If not, an alternative model should be considered. This is our chief goal in this paper. In particular, we present tests of the null hypothesis that the beta model is correctly specified against the alternative hypothesis that it is misspecified. Alternative models should be considered for the application at hand whenever the null hypothesis of correct beta model specification is rejected. In particular, we consider a general test of correct model specification that was introduced by [11], known as ‘the information matrix test’, and also some variants of it. The name of the test stems from the fact that the information matrix equality is known to only hold when the model is correctly specified. Information matrix test statistics are based on the sample counterparts of the model matrices that comprise such an equality. They were derived for several statistical models and distributions, e.g., the Gaussian linear regression model (see [12]), binary data models (see [13]), linear regressions with autoregressive and moving average errors (see [14]), logistic regressions (see [15]), beta-binomial models (see [16]), and the negative binomial law (see [17]).

We obtain three information matrix test statistics for testing the null hypothesis that the beta model is correctly specified. They differ in the estimator used for the covariance matrix of a given random vector. The first two test statistics employ different estimators of the random vector’s asymptotic covariance matrix whereas the third and final test statistic employs a resampling-based estimator of its exact covariance matrix. Since our numerical results show that the first two tests are considerably size-distorted in small to moderately large sample sizes, we also perform them using bootstrap critical values. It is noteworthy that the tests we develop are based on the information equality, which only holds when the model specification is not in error. As a consequence, they have power against any form of model misspecification, not only of distributional nature.

The Monte Carlo simulation evidence we report shows that the tests perform well, especially when coupled with bootstrap resampling. As noted above, three variants of the information matrix test are considered. For two of them, bootstrap resampling is used to obtain critical values that do not rely on asymptotic approximations whereas, in the remaining test, bootstrap resampling is used to estimate a covariance matrix that is used in the test statistic. Overall, the use of bootstrap resampling yields good control of the type I error frequency. Simulations in which the data were generated under the alternative hypothesis show that the tests are typically able to detect incorrect model specification, especially when the sample size is not small. Consider, e.g., the Kumaraswamy distribution, which is commonly used as an alternative law for fractional data. The numerical results we report show that when such a law is the true data-generating mechanism, the information matrix tests reject the beta model with probabilities around 0.9 for samples that contain 250 data points at the 10% significance level. Our Monte Carlo evidence also shows that the tests can successfully reject the univariate beta model when the sample size is not very small and the underlying law is beta but with non-constant means.

We model state and county Covid-19 mortality rates in the United States (US) using the univariate beta model. Three sample periods are considered: the first only includes observations from prior to the start of the nationwide vaccination campaign, the second encompasses data obtained before and after such a date, and the third and final period only includes data collected when the vaccination drive was under way. The testing inferences suggest that the beta law yields an adequate data representation for Covid-19 death rates in the first and third time periods. By contrast, the beta law is rejected when the data are heterogeneous, i.e., when the mortality rates are computed using information gathered prior to and during the nationwide vaccination drive. Interestingly, the univariate beta model is found to adequately describe the data in the third time period, in which mortality rates are negatively impacted by the reach of the vaccination drive. This happens because (i) in the initial part of the sample period vaccination was incipient and had little impact on the overall mortality figures and (ii) the negative relationship between the two variables is weakened by a few states, namely: Alaska, Arizona, Florida, Massachusetts, North Dakota, and Rhode Island. When all counties in such states are removed from the data, the inverse relationship between vaccination reach and death rates become considerably more intense, and the information matrix tests reject the adequacy of the univariate beta model, thus indicating that a more elaborate model should be used. The information matrix tests’ inferences thus indicate that as long as the negative impact of vaccination reach on death rates is moderate, the beta law can be used to represent Covid-19 mortality rates. When such a negative impact becomes more pronounced, the univariate beta model should no longer be used.

The remainder of the paper is organized as follows. The beta distribution and the corresponding maximum likelihood parameter estimation are briefly presented in the next section. In the third section, information matrix misspecification tests for the beta model are obtained. In particular, we introduce five tests, three of which based on bootstrap resampling. Monte Carlo simulation results are presented in the fourth section. We evaluate the tests’ null (size) and non-null (power) behaviors. An empirical analysis of Covid-19 mortality rates in the US is presented and discussed in the fifth section. Finally, concluding remarks are offered in the sixth section together with directions for future research.

## The beta distribution

Let *Y* be a beta-distributed random variable. Its density function, following the parametrization introduced by [18], can be expressed as

$$\begin{array}{c} f\left(y;\mu ,\phi \right)=\frac{\Gamma (\phi )}{\Gamma (\mu \phi )\Gamma ((1-\mu )\phi )}y^{\mu \phi -1}{\left(1-y\right)}^{(1-\mu )\phi -1},\;0<y<1,0<\mu <1,\phi >0, \end{array}$$ (1)

where IE(Y)=μandϕ and *ϕ* is a precision parameter since, for fixed *μ*, Var(*Y*) = *μ*(1 − *μ*)/(1 + *ϕ*) decreases as *ϕ* increases. We write Y∼ℬ(μ,ϕ). Unlike the standard beta parametrization, the parameters in (1) can be directly interpreted in terms of the distribution mean and precision. As we will see in the fifth section, it is useful to compare estimated precisions obtained from different model fits. The beta density in (1) is symmetric if *μ* = 0.5 and asymmetric otherwise, and it reduces to the uniform density if *μ* = 0.5 and *ϕ* = 2. The beta density can be asymmetric to the left or to the right, and it can also be J-shaped, inverted J-shaped, and U-shaped. It is thus clear, as noted by [6], that “[b]eta distributions are very versatile and a variety of uncertainties can be usefully modelled by them.” It is noted that “[t]his flexibility encourages its empirical use in a wide range of applications.”

Let *Y*_1_, …, *Y*_*n*_ be independent and identically distributed (i.i.d.) beta-distributed random variables and let *y*_1_, …, *y*_*n*_ be their observed, realized values. In what follows, ***Y*** and ***y*** denote the *n*-vectors of such random variables and realizations, respectively. Also, ***θ*** = (*μ*, *ϕ*)^⊤^ is the vector of beta parameters. Whenever required, we refer to *μ* and *ϕ* as *θ*_1_ and *θ*_2_, respectively. The log-likelihood function for ***Y*** evaluated at ***y*** is

$$\begin{array}{c} \ell \left(\mu ,\phi ;y\right)\equiv \ell \left(\theta ;y\right)=\sum_{t=1}^{n}\ell \left(\theta ;y_{t}\right), \end{array}$$

where *ℓ*(***θ***; *y*_*t*_) = log(*f*(*y*_*t*_; *μ*, *ϕ*)) is the *t*th individual log-likelihood, which is given by

$$\begin{array}{c} \ell \left(\theta ;y_{t}\right)=\text{log}\left(\Gamma \left(\phi \right)\right)-\text{log}\left(\Gamma \left(\mu \phi \right)\right)-\text{log}\left(\Gamma \left(\left(1-\mu \right)\phi \right)\right)+\left(\mu \phi -1\right)y_{t}^{*}+\left(\phi -2\right)y_{t}^{†}, \end{array}$$

with $y_{t}^{*}=\text{log}(y_{t}/(1-y_{t}))$ and $y_{t}^{†}=\text{log}(1-y_{t})$. Let $Y_{t}^{*}=\text{log}(Y_{t}/(1-Y_{t}))$, $\mu^{*}=I\;E(Y_{t}^{*})$, $Y_{t}^{†}=\text{log}(1-Y_{t})$ and $\mu^{†}=I\;E(Y_{t}^{†})$. It follows that μ* = *ψ*(*μϕ*) − *ψ*((1 − *μ*)*ϕ*) and *μ*^†^ = *ψ*((1 − *μ*)*ϕ*) − *ψ*(*ϕ*), where *ψ* is the digamma function, i.e., the first derivative of the logarithm of the gamma function.

The score vector is ∇*ℓ*(***θ***, ***y***) = ∂*ℓ*(***θ***;***y***)/∂***θ*** = (∂*ℓ*(***θ***;***y***)/∂ *μ*, ∂*ℓ*(***θ***;***y***)/∂ *ϕ*)^⊤^, where

$$\begin{array}{c} \frac{\partial \ell (\theta ;y)}{\partial \mu }=\sum_{t=1}^{n}\phi \left(y_{t}^{*}-\mu^{*}\right)\;\text{and}\;\frac{\partial \ell (\theta ;y)}{\partial \phi }=\sum_{t=1}^{n}\mu \left(y_{t}^{*}-\mu^{*}\right)+\left(y_{t}^{†}-\mu^{†}\right). \end{array}$$

Fisher’s information matrix for a single observation, *B*(***θ***), is defined as the expected value of the individual log-likelihood derivative outer product: $B(\theta )=\text{I}\;\text{E}(\partial l(\theta ;Y_{t})/\partial \theta \times \partial l(\theta ;Y_{t})/\partial \theta^{⊤})$. For the beta model,

$$\begin{array}{c} B\left(\theta \right)=[\begin{array}{cc} B_{\mu \mu } & B_{\mu \phi }\\ B_{\phi \mu } & B_{\phi \phi } \end{array}], \end{array}$$

where *B*_*μμ*_ = *ϕ*^2^*w*, *B*_*μϕ*_ = *B*_*μϕ*_ = *c* and *B*_*ϕϕ*_ = (*μc*)/*ϕ* + (1 − *μ*)*ψ*′((1 − *μ*)*ϕ*) − *ψ*′(*ϕ*), *ψ*′ being the trigamma function. The expressions for the quantities *w* and *c* can be found in the Appendix. The total information matrix, i.e., the information matrix for the complete sample, is *nB*(***θ***).

The maximum likelihood estimator of ***θ***, say $\hat{\theta }$, cannot be expressed in closed-form. Parameter estimates are typically obtained by numerically maximizing the log-likelihood function using a Newton or quasi-Newton nonlinear optimization algorithm. In what follows, we will use Broyden-Fletcher-Goldfarb-Shanno (BFGS) algorithm with analytical first derivatives for maximum likelihood estimation; for details, see [19].

## Beta misspecification tests

Our goal in what follows is to obtain tests of correct model specification for the beta distribution. Our focus is on the information matrix test introduced in full generality by [11]. Let ***θ***_0_ = (*μ*_0_, *ϕ*_0_)^⊤^ be the true parameter value. The beta model is taken to be correctly specified if *Y*_*t*_ follows the beta law with parameter vector ***θ***_0_ ∀ *t*.

Let $A(\theta )=\text{I}\;\text{E}(\partial^{2}l(\theta ;Y_{t})/\partial \theta \partial \theta^{⊤})$ be the expected Hessian of *ℓ*(***θ***; *Y*_*t*_). When the model is correctly specified and under the assumptions listed in Sections 2 and 3 of [11], the information matrix equality holds: *B*(***θ***_0_) = − *A*(***θ***_0_); alternatively, *A*(***θ***_**0**_) + *B*(***θ***_0_) = 0. Evidence that such an equality fails to hold is thus taken as evidence of incorrect model specification. Our interest lies in testing the null hypothesis $H_{0}:A(\theta_{0})+B(\theta_{0})=0$ (correct beta model specification) against the alternative hypothesis $H_{1}:A(\theta_{0})+B(\theta_{0})\neq 0$ (beta model misspecification).

In what follows, we will present three information matrix test statistics that can be used to test the correct beta model specification. At the outset, we derive several quantities that are used in such test statistics. We obtain, for the beta model,

$$\begin{array}{c} A_{n}\left(\theta ;Y\right)=\frac{1}{n}\sum_{t=1}^{n}\frac{\partial^{2}\ell \left(\theta ;Y_{t}\right)}{\partial \theta \partial \theta^{⊤}}=\frac{1}{n}\sum_{t=1}^{n}[\begin{array}{cc} A_{n_{\mu \mu }} & A_{n_{\phi \mu }}\\ A_{n_{\mu \phi }} & A_{n_{\phi \phi }} \end{array}], \end{array}$$

where $A_{n_{\mu \mu }}=-\phi^{2}w$, $A_{n_{\phi \mu }}=A_{n_{\mu \phi }}=(Y_{t}^{*}-\mu^{*})-c$ and $A_{n_{\phi \phi }}=-(\mu c)/\phi -(1-\mu )\psi^{\prime }((1-\mu )\phi )+\psi^{\prime }(\phi )$. Expressions for *c* and *w* can be found, as noted earlier, in the Appendix. Additionally,

$$\begin{array}{c} B_{n}\left(\theta ;Y\right)=\frac{1}{n}\sum_{t=1}^{n}\frac{\partial \ell (\theta ;Y_{t})}{\partial \theta }\times \frac{\partial \ell (\theta ;Y_{t})}{\partial \theta^{⊤}}=\frac{1}{n}\sum_{t=1}^{n}[\begin{array}{cc} B_{n_{\mu \mu }} & B_{n_{\phi \mu }}\\ B_{n_{\mu \phi }} & B_{n_{\phi \phi }} \end{array}], \end{array}$$

where $B_{n_{\mu \mu }}=\phi^{2}(Y_{t}^{*}-\mu^{*}{)}^{2}$, $B_{n_{\phi \mu }}=B_{n_{\mu \phi }}=\phi \left(Y_{t}^{*}-\mu^{*}\right)\left[\mu (Y_{t}^{*}-\mu^{*})+(Y_{t}^{†}-\mu^{†})\right]$ and $B_{n_{\phi \phi }}={\left[\mu (Y_{t}^{*}-\mu^{*})+(Y_{t}^{†}-\mu^{†})\right]}^{2}$. Notice that *A*_*n*_(***θ***;***Y***) and *B*_*n*_(***θ***;***Y***) evaluated at $\theta =\hat{\theta }$ are consistent estimators of *A*(***θ***_0_) and *B*(***θ***_0_), respectively.

We also need to obtain

$$\begin{array}{c} D_{n}\left(\theta \right)\equiv D_{n}\left(\theta ;Y\right)=\frac{1}{n}\sum_{t=1}^{n}d\left(\theta ;Y_{t}\right), \end{array}$$

where

$$\begin{array}{c} d\left(\theta ;Y_{t}\right)=\text{vech}(\frac{\partial^{2}\ell \left(\theta ;Y_{t}\right)}{\partial \theta \partial \theta^{⊤}}+\frac{\partial \ell (\theta ;Y_{t})}{\partial \theta }\times \frac{\partial \ell (\theta ;Y_{t})}{\partial \theta^{⊤}}) \end{array}$$

is a 3 × 1 vector with *l*th component given by

$$\begin{array}{c} d_{l}\left(\theta ;Y_{t}\right)=\frac{\partial^{2}\ell \left(\theta ;Y_{t}\right)}{\partial \theta_{i}\partial \theta_{j}}+\frac{\partial \ell (\theta ;Y_{t})}{\partial \theta_{i}}\times \frac{\partial \ell (\theta ;Y_{t})}{\partial \theta_{j}}, \end{array}$$

with *i* = *j* = 1 for *l* = 1; *i* = 1 and *j* = 2 for *l* = 2; *i* = *j* = 2 for *l* = 3. For the beta distribution, we obtain

$$\begin{array}{cc} d_{1}\left(\theta ;Y_{t}\right) & =\phi^{2}\left[{\left(Y_{t}^{*}-\mu^{*}\right)}^{2}-w\right],\\ d_{2}\left(\theta ;Y_{t}\right) & =\left(Y_{t}^{*}-\mu^{*}\right)-c+\phi \left(Y_{t}^{*}-\mu^{*}\right)[\mu \left(Y_{t}^{*}-\mu^{*}\right)+\left(Y_{t}^{†}-\mu^{†}\right)],\\ d_{3}\left(\theta ;Y_{t}\right) & =-\mu \frac{c}{\phi }-\left(1-\mu \right)\psi^{\prime }\left(\left(1-\mu \right)\phi \right)+\psi^{\prime }\left(\phi \right)+{\left[\mu \left(Y_{t}^{*}-\mu^{*}\right)+\left(Y_{t}^{†}-\mu^{†}\right)\right]}^{2}. \end{array}$$

Note that **D**_*n*_(***θ***;***Y***) = vech(*A*_*n*_(***θ***;***Y***) + *B*_*n*_(***θ***;***Y***)) is a vector that contains three elements. The information matrix test statistics we consider are functions of such a restrictions vector evaluated at $\theta =\hat{\theta }$.

Let

$$\begin{array}{cc} V(\theta )=I\;E & [(d\left(\theta ;Y_{t}\right)-\nabla D\left(\theta \right)A{\left(\theta \right)}^{-1}\nabla \ell \left(\theta ;Y_{t}\right))\times \\ & {\left(d\left(\theta ;Y_{t}\right)-\nabla D\left(\theta \right)A{\left(\theta \right)}^{-1}\nabla \ell \left(\theta ;Y_{t}\right)\right)}^{⊤}], \end{array}$$

where $D(\theta )=\text{I}\;\text{E}(d(\theta ;Y_{t}))$ and ∇***D***(***θ***) = ∂***D***(***θ***)/∂***θ***^⊤^. [11] showed that, under correct model specification, $\sqrt{n}D_{n}(\hat{\theta };Y)$ is asymptotically normally distributed with zero mean and covariance matrix *V*(***θ***_0_) and noticed that a natural consistent estimator for *V*(***θ***_0_) is

$$\begin{array}{cc} V_{n1}\left(\theta \right)=\frac{1}{n}\sum_{t=1}^{n} & [(d\left(\theta ;Y_{t}\right)-\nabla D_{n}\left(\theta ;Y\right)A_{n}{\left(\theta ;Y\right)}^{-1}\nabla \ell \left(\theta ;Y_{t}\right))\times \\ & {\left(d\left(\theta ;Y_{t}\right)-\nabla D_{n}\left(\theta ;Y\right)A_{n}{\left(\theta ;Y\right)}^{-1}\nabla \ell \left(\theta ;Y_{t}\right)\right)}^{⊤}] \end{array}$$

evaluated at $\theta =\hat{\theta }$, where ∇***D***_*n*_(***θ***;***Y***) = ∂***D***_*n*_(***θ***;***Y***)/∂***θ***^⊤^. Closed-form expressions for the elements of ∇**D**_*n*_(***θ***;***Y***) in the beta model are given in the Appendix.

The first information matrix test statistic is

$$\begin{array}{c} \zeta_{1}=nD_{n}{\left(\hat{\theta }\right)}^{⊤}{\left(V_{n1}\left(\hat{\theta }\right)\right)}^{-1}D_{n}\left(\hat{\theta }\right), \end{array}$$

where *q* is the number of components of **D**_*n*_(***θ***;***Y***) considered (*q* ≤ 3). Under $H_{0}$, *ζ*_1_ is asymptotically distributed as $\chi_{q}^{2}$. The test is then carried out using critical values from such a distribution, i.e., $H_{0}$ is rejected at significance level *α* ∈ (0, 1) if $\zeta_{1}>\chi_{q,1-\alpha }^{2}$, where $\chi_{q,1-\alpha }^{2}$ is the 1 − *α* $\chi_{q}^{2}$ quantile.

Alternative information matrix test statistics can be obtained by considering different consistent estimators for *V*(***θ***_0_). [20, 21] showed that it is possible to use a covariance matrix estimator that does not require third order log-likelihood derivatives. They use the fact that, under $H_{0}$, $\nabla D(\theta_{0})=-\text{I}\;\text{E}(d(\theta_{0};Y_{t})\times \nabla l{\left(\theta_{0};Y_{t}\right)}^{⊤})$; see [21]. Let

$$\begin{array}{c} L_{n}\left(\theta ;Y\right)=-\frac{1}{n}\sum_{t=1}^{n}d\left(\theta ;Y_{t}\right)\times \nabla \ell {\left(\theta ;Y_{t}\right)}^{⊤}. \end{array}$$

The Chesher-Lancaster estimator of *V*(***θ***_0_) is

$$\begin{array}{cc} V_{n2}\left(\theta \right)=\frac{1}{n}\sum_{t=1}^{n} & [(d\left(\theta ;Y_{t}\right)+L_{n}\left(\theta ;Y\right)B_{n}{\left(\theta ;Y\right)}^{-1}\nabla \ell \left(\theta ;Y_{t}\right))\times \\ & {\left(d\left(\theta ;Y_{t}\right)+L_{n}\left(\theta ;Y\right)B_{n}{\left(\theta ;Y\right)}^{-1}\nabla \ell \left(\theta ;Y_{t}\right)\right)}^{⊤}] \end{array}$$

evaluated at $\theta =\hat{\theta }$. The corresponding information matrix test statistic is

$$\begin{array}{c} \zeta_{2}=nD_{n}{\left(\hat{\theta }\right)}^{⊤}{\left(V_{n2}\left(\hat{\theta }\right)\right)}^{-1}D_{n}\left(\hat{\theta }\right). \end{array}$$

Under $H_{0}$, *ζ*_2_ is asymptotically distributed as $\chi_{q}^{2}$ and, as before, the test is carried out using asymptotic critical values.

It is noteworthy that $V_{n1}(\hat{\theta })$ and $V_{n2}(\hat{\theta })$ are consistent estimators of *V*(***θ***_0_), the latter being the asymptotic covariance matrix of $\sqrt{n}D_{n}(\hat{\theta };Y)$. A consistent estimator of the exact covariance matrix of such a vector, say $V_{S_{n}}(\theta_{0})$, can be obtained by using parametric bootstrap resampling, as shown by [22]. The bootstrap estimator of $V_{S_{n}}(\theta_{0})$ based on *B* bootstrap samples can be computed as follows:

1. Using the original sample ***Y*** = (*Y*_1_, …, *Y*_*n*_)^⊤^, compute $\hat{\theta }$.
2. Obtain a random sample of size *n*, say $Y_{b}^{*}=(Y_{1}^{*},\ldots ,Y_{n}^{*}{)}^{⊤}$, from the beta law with ***θ*** replaced with $\hat{\theta }$, i.e., perform the pseudo-data generation from $f(\cdot ;\hat{\theta })$.
3. Using $Y_{b}^{*}$, compute ${\hat{\theta }}_{b}^{*}$ and $D_{n}({\hat{\theta }}_{b}^{*};Y_{b}^{*})$.
4. Execute steps (2) and (3) *B* times, where *B* is a large positive integer.
5. Using the bootstrap replicates $D_{n}({\hat{\theta }}_{1}^{*};Y_{1}^{*}),\ldots ,D_{n}({\hat{\theta }}_{B}^{*};Y_{B}^{*})$, compute the bootstrap estimator of $V_{S_{n}}(\theta_{0})$ as
  $$\begin{array}{c} {\hat{V}}_{n3,B}^{*}=\frac{n}{B-1}\sum_{b=1}^{B}\left(D_{n}\left({\hat{\theta }}_{b}^{*};Y_{b}^{*}\right)-\bar{D}\right){\left(D_{n}\left({\hat{\theta }}_{b}^{*};Y_{b}^{*}\right)-\bar{D}\right)}^{⊤}, \end{array}$$
  where $\bar{D}=B^{-1}\sum_{b=1}^{B}D_{n}({\hat{\theta }}_{b}^{*};Y_{b}^{*})$.

For fixed *n* and as *B* → ∞, it follows that ${\hat{V}}_{n3,B}^{*}\overset{p}{\to }V_{S_{n}}(\hat{\theta })$; see [22]. We thus arrive at a third information matrix test statistic for testing the correct beta model specification. It is given by

$$\begin{array}{c} \zeta_{3}=nD_{n}{\left(\hat{\theta }\right)}^{⊤}{\left(V_{n3,B}^{*}\right)}^{-1}D_{n}\left(\hat{\theta }\right). \end{array}$$

Under $H_{0}$, for fixed *B* and *n* → ∞, *ζ*_3_ is asymptotically distributed as $T_{q,B-1}^{2}$, i.e., as Hotelling’s *T*-squared distribution with *q* and *B* − 1 degrees of freedom; see [22]. As before, the test is performed using asymptotic critical values.

The information matrix test statistics *ζ*_1_, *ζ*_2_ and *ζ*_3_ measure the sample evidence against the correct beta model specification. When they assume large values and $H_{0}$ is rejected at the usual significance levels, an alternative model should be used. A word of caution, however, is in order. The test based on *ζ*_3_ is expected to perform well in small to moderately large samples since the test statistic uses a bootstrap estimator of the exact covariance matrix of $\sqrt{n}D_{n}(\hat{\theta };Y)$. The tests based on *ζ*_1_ and *ζ*_2_, by contrast, may be considerably size-distorted when *n* is not large since the test statistics use estimators of the asymptotic covariance matrix of $\sqrt{n}D_{n}(\hat{\theta };Y)$ and such an asymptotic covariance matrix may be a poor approximation for its exact counterpart when *n* is not large. To remedy that, we recommend that *ζ*_1_ and *ζ*_2_ testing inferences be based on critical values obtained from bootstrap resampling instead of on $\chi_{q,1-\alpha }^{2}$ (asymptotic critical values). To that end, for *i* = 1, 2:

1. Using the original sample ***Y*** = (*Y*_1_, …, *Y*_*n*_)^⊤^, compute $\hat{\theta }$ and *ζ*_*i*_.
2. Obtain a random sample of size *n*, say $Y_{b}^{*}=(Y_{1}^{*},\ldots ,Y_{n}^{*}{)}^{⊤}$, from the beta law with ***θ*** replaced with $\hat{\theta }$.
3. Using $Y_{b}^{*}$, compute ${\hat{\theta }}_{b}^{*}$ and $\zeta_{i,b}^{*}$.
4. Execute steps (2) and (3) *B* times.
5. Reject $H_{0}$ at significance level *α* if *ζ*_*i*_ exceeds the 1 − *α* quantile of $\zeta_{i,1}^{*},\ldots ,\zeta_{i,B}^{*}$.

The use of bootstrap resampling when performing testing inferences based on the information matrix test statistics *ζ*_1_ and *ζ*_2_ may considerably reduce size distortions since the critical values used in such tests are now obtained from estimates of the test statistics’ exact null distributions.

As noted earlier, it is possible to test *q* ≤ 3 restrictions. In what follows, we will test two restrictions since numerical evaluations not shown here for brevity revealed that the third element of $D_{n}(\hat{\theta };Y)$ always assumes very small values and has very small variance, especially when dispersion is low, which renders near singular estimates of *V*(***θ***_0_). As noted by [11], when an indicator is identically null it should be ignored; see the example on page 10 of his article. Unlike what happens in his example, the maximum likelihood estimators in our case cannot be expressed in closed form, and that is why we had to resort to numerical evaluations to determine whether there is a non-relevant restriction. We thus test *q* = 2 restrictions by using **d**(***θ***; *Y*_*t*_) = (*d*_1_(***θ***; *Y*_*t*_), *d*_2_(***θ***; *Y*_*t*_)))^⊤^. Correspondingly, we drop the last row of ∇***D***_*n*_(***θ***;***Y***). The asymptotic null distribution of *ζ*_1_ and *ζ*_2_ is $\chi_{2}^{2}$, and that of *ζ*_3_ is $T_{2,B-1}^{2}$, where *B* is the number of bootstrap replications used in the estimation of $V_{S_{n}}(\theta_{0})$.

According to [11], it is expected that the tests will be consistent (i.e., have unit power asymptotically) against any alternative which renders the usual maximum likelihood inference techniques invalid. In our case, maximum likelihood inference involves the estimation of the beta distribution mean and precision parameters. When *Y* follows other laws or when the values of the beta parameters are not the same for all observations, the test statistics are expected to diverge in probability so that unit power is achieved asymptotically. We performed Monte Carlo simulations using a number of alternative models as the true data generating mechanism, which include alternative laws, data inflation, and neglected regression structure. The results from these simulations are presented in the next section. They show evidence of asymptotic unit power under all sources of model misspecification we considered.

## Numerical evidence

We will now numerically evaluate the performance of the information matrix tests when used to determine whether the beta distribution yields a satisfactory data fit, i.e., when used to determine whether the beta model is correctly specified. Data generation is carried out under the null and alternative hypotheses (correct and incorrect model specification, respectively). Beta random number generation is performed using the acceptance-rejection method based on uniform random draws obtained using the Mersenne Twister method. Parameter estimates are obtained by numerically maximizing the beta log-likelihood function using the BFGS quasi-Newton algorithm with analytical first derivatives. The starting values used in the estimation of *μ* and *ϕ* are, respectively, $\bar{y}$ and $\bar{y}(1-\bar{y})/\overset{⌃}{\text{Var}}(Y)-1$, where $\bar{y}=n^{-1}\sum_{t=1}^{n}y_{t}$ and $\overset{⌃}{\text{Var}}(Y)=(n-1{)}^{-1}\sum_{t=1}^{n}(y_{t}-\bar{y}{)}^{2}$. The number of Monte Carlo and bootstrap replications are, respectively, 5000 and 500. The null hypothesis is $H_{0}:$ “the beta model is correctly specified” and the alternative hypothesis is $H_{1}:$ “the beta model is misspecified”.

The following tests are performed: *ζ*_1_, *ζ*_1*B*_, *ζ*_2_, *ζ*_2*B*_, and *ζ*_3_. The *ζ*_1*B*_ and *ζ*_2*B*_ tests employ bootstrap critical values, and the *ζ*_3_ test statistic uses a bootstrap covariance matrix estimate. The simulations were performed using the R statistical computing environment; see [23].

At the outset, data generation is carried out under $H_{0}$, i.e., the observations are obtained as random draws from the beta distribution with mean *μ* and precision *ϕ*. The significance levels and sample sizes are, respectively, *α* = 10%, 5%, 1% and *n* = 50, 100, 250, 500, 1000, 5000.

In what follows, we will report the tests’ null and non-null rejection rates obtained from size (data generated under $H_{0}$) and power (data generated under $H_{1}$) simulations, respectively. Additionally, we will present *p*-value plots and size-power plots for the *ζ*_1_, *ζ*_2_ and *ζ*_3_ tests, i.e., for the tests that do not employ bootstrap critical values. Based on the size simulations (the data-generating process is beta), we plot the tests’ empirical sizes (vertical axis) against nominal sizes, i.e., against values of *α* ∈ (0, 1) (horizontal axis). The 45° line indicates perfect agreement between actual and nominal sizes. Curves that lie above (below) such a diagonal line for a given range of values of *α* are indicative of liberal (conservative) behavior at those significance levels. It should be noted that, in this graphical analysis, *α* is not fixed at three values (0.10, 0.05 and 0.01) but varies from close to zero up to close to one. We thus obtain a comprehensive view of the tests’ null behaviors. We also present plots that relate the tests’ empirical powers (vertical axis) to the corresponding sizes (horizontal axis), computed for values of *α* ranging from close to zero up to close to one. The non-null rejection rates are computed using a data-generating process that differs from the beta law. It should be noted that since the non-null rejection rates are plotted using the empirical critical value for each nominal size (and not using asymptotic critical values) it is possible to compare the tests’ non-null behaviors by properly accounting for any existing size distortions. The higher the curve, the more powerful the test. For more details on these plots, see [24].

In the first size simulation, the data are generated from the beta law with *μ* = 0.2 and *ϕ* = 20, 40, 80, 120. The null rejection rates of the *ζ*_1_, *ζ*_1*B*_, *ζ*_2_, *ζ*_2*B*_ and *ζ*_3_ tests are shown in Table 1. All entries are percentages. The reported results lead to interesting conclusions. First, the *ζ*_1_ and *ζ*_2_ tests, which use asymptotic critical values, are quite liberal when the sample size is not very large; even with *n* = 1000, considerable size distortions take place. Second, such tests have effective sizes that are close to the nominal sizes when bootstrap (rather than asymptotic) critical values are used. For example, when *ϕ* = 40 and *n* = 100, the sizes of *ζ*_1_ and *ζ*_2_, at *α* = 10%, are 17.6% and 37.4%; when bootstrap critical values are used, these rates drop to 10.7% and 9.8%, respectively. The use of bootstrap resampling thus considerably reduces size distortions. Third, the size distortions of *ζ*_1_ decrease when the value of *ϕ* increases. For example, the test’s null rejection rates for *n* = 100 and *α* = 10% are 20.1% and 12.3% when *ϕ* = 20 and *ϕ* = 120, respectively. It is worth noticing that the variance of *Y* decreases when the value of *ϕ* increases, and that translates into more accurate testing inferences. Fourth, the *ζ*_3_ test tends to be conservative when *n* ≤ 1000, and displays null rejection rates close to the nominal levels with *n* = 5000.

**Table 1:** Null rejection rates (%), *μ* = 0.2.

*n*	*ζ*_1_	*ζ*_1*B*_	*ζ*_2_	*ζ*_2*B*_	*ζ*_3_	*ζ*_1_	*ζ*_1*B*_	*ζ*_2_	*ζ*_2*B*_	*ζ*_3_
	*ϕ* = 20					*ϕ* = 40				
	*α* = 10%									
50	17.5	9.9	48.6	9.5	5.5	15.4	10.0	50.1	10.0	4.2
100	20.1	11.0	39.1	10.7	6.1	17.6	10.7	37.4	9.8	5.2
250	19.0	11.0	27.9	10.8	7.1	17.0	9.9	27.1	10.2	6.6
500	17.1	10.6	21.3	10.8	8.2	15.8	10.4	20.9	10.5	7.4
1000	14.8	10.1	17.3	9.9	9.1	14.0	10.2	17.1	10.4	8.6
5000	11.9	10.2	12.3	10.3	9.9	11.6	10.4	12.2	10.2	9.7
	*α* = 5%									
50	10.1	5.3	41.8	4.8	3.3	9.2	5.3	43.7	5.2	2.5
100	12.4	5.7	31.9	5.5	3.5	10.6	5.3	30.7	5.4	3.0
250	12.6	5.3	21.3	5.4	4.0	10.4	4.9	20.7	5.3	3.7
500	11.1	5.6	15.7	5.5	4.5	9.9	5.5	15.2	5.6	4.1
1000	9.1	5.3	11.7	5.4	4.8	8.5	5.3	11.3	5.4	4.7
5000	6.8	5.5	7.2	5.6	4.9	6.5	5.3	7.1	5.4	4.9
	*α* = 1%									
50	3.8	1.1	30.2	1.2	1.2	3.5	1.0	31.6	1.3	0.9
100	4.7	1.4	22.4	1.4	1.4	3.9	1.1	20.8	1.2	0.9
250	4.6	1.0	12.9	1.3	1.5	3.9	1.2	11.9	1.4	1.3
500	4.8	1.1	8.2	1.4	1.5	3.9	1.1	8.0	1.2	1.3
1000	3.7	1.6	5.2	1.6	1.5	3.4	1.3	5.2	1.5	1.6
5000	2.3	1.5	2.5	1.4	1.2	1.8	1.2	2.2	1.2	0.8
	*ϕ* = 80					*ϕ* = 120				
	*α* = 10%									
50	13.8	9.9	48.5	10.4	4.3	12.8	10.6	49.0	10.3	4.1
100	14.0	9.8	37.8	10.1	4.5	12.3	10.2	37.7	10.2	4.7
250	14.5	10.1	26.5	9.5	5.8	12.7	10.3	25.7	10.7	5.2
500	14.0	10.2	20.9	9.7	7.0	12.4	9.9	21.0	10.2	6.2
1000	13.4	10.6	17.6	10.6	7.9	12.6	10.8	17.0	10.7	7.6
5000	11.1	9.9	12.0	9.9	9.5	11.0	10.1	12.2	9.9	9.4
	*α* = 5%									
50	7.8	4.8	41.5	4.8	2.5	7.7	5.4	42.1	5.3	2.2
100	8.1	5.0	30.7	5.0	2.4	7.3	4.9	30.9	5.3	2.6
250	8.6	5.2	20.7	4.9	3.2	8.0	6.0	19.7	5.8	2.9
500	8.3	5.1	14.6	4.9	3.9	7.1	5.0	14.6	5.3	3.0
1000	7.8	5.3	11.6	4.9	4.1	7.1	5.5	11.5	5.7	3.8
5000	6.3	5.3	7.1	5.2	4.6	5.5	4.9	6.4	5.0	4.4
	*α* = 1%									
50	2.6	1.2	29.3	1.5	0.9	3.0	1.2	30.1	1.3	0.6
100	3.0	1.2	20.1	1.2	0.8	2.5	1.1	21.1	1.2	0.9
250	2.9	1.3	11.3	1.1	0.9	3.1	1.6	12.1	1.7	1.1
500	2.7	1.0	7.4	1.0	1.3	2.3	1.4	7.6	1.4	0.8
1000	2.6	1.2	4.7	1.1	1.2	2.6	1.5	5.5	1.3	0.9
5000	1.8	1.1	2.2	1.1	1.1	1.3	1.0	1.7	0.9	0.9

In the second set of size simulations, data generation was performed from the beta distribution with *μ* = 0.5 and the same precision values as before. The tests’ null rejection rates are presented in Table 2. All entries are percentages. In general, the new results are similar to those in the previous scenario. The *ζ*_1_ and *ζ*_2_ tests remain liberal, with *ζ*_1_ exhibiting considerably higher null rejection rates relative to previous results. For example, when *ϕ* = 40, *α* = 10% and *n* = 100, the null rejection rate of *ζ*_1_ is 28.4% whereas in the previous scenario it was 17.6%. The testing inferences are less accurate here because there exists more uncertainty since the variance of the beta distribution is maximal when *μ* = 0.5; recall that such a variance is *μ*(1− *μ*)/(1 + *ϕ*). The figures in Table 2 further show that the *ζ*_1*B*_ and *ζ*_2*B*_ tests display the smallest size distortions, being accurate even when *n* is small. For example, when *ϕ* = 20 and *n* = 50, the sizes of *ζ*_1*B*_ and *ζ*_2*B*_, at *α* = 10%, are 10.0% and 9.6%, respectively. It is thus clear that bootstrap resampling works remarkably well. Additionally, the *ζ*_3_ test remains conservative when *μ* = 0.5, but only for *α* = 10% and 5%. The test exhibits small size distortions when *n* ≥ 250. For instance, when *ϕ* = 20 and *n* = 250, the test’s null rejection rate, at *α* = 10%, is 9.3%.

**Table 2:** Null rejection rates (%), *μ* = 0.5.

*n*	*ζ*_1_	*ζ*_1*B*_	*ζ*_2_	*ζ*_2*B*_	*ζ*_3_	*ζ*_1_	*ζ*_1*B*_	*ζ*_2_	*ζ*_2*B*_	*ζ*_3_
	*ϕ* = 20					*ϕ* = 40				
	*α* = 10%									
50	30.5	10.0	49.1	9.6	6.9	31.2	9.6	49.8	10.3	7.1
100	28.2	10.5	38.2	10.8	8.0	28.4	10.9	38.7	10.7	8.3
250	23.2	11.2	27.3	10.8	9.3	21.8	9.6	26.2	9.5	8.5
500	17.6	9.4	19.8	9.3	8.3	19.3	10.9	21.5	10.8	8.9
1000	15.7	10.2	16.8	10.1	9.5	16.1	10.9	17.1	10.8	9.7
5000	12.1	10.2	12.4	10.2	10.3	12.1	10.5	12.3	10.4	9.7
	*α* = 5%									
50	22.1	4.9	42.2	4.7	4.8	22.1	5.0	42.1	5.1	4.5
100	20.0	5.4	31.9	5.4	5.0	20.3	5.5	32.2	5.0	5.1
250	16.4	6.0	21.1	6.1	5.3	14.9	4.8	19.4	4.6	4.9
500	12.0	4.8	14.4	4.8	4.8	12.7	5.6	15.2	5.8	5.1
1000	10.2	5.3	11.0	5.4	4.8	10.8	5.6	11.8	5.6	5.1
5000	6.6	5.4	6.9	5.4	5.4	6.4	4.9	6.6	4.9	5.1
	*α* = 1%									
50	9.7	1.4	30.3	1.1	2.5	9.4	1.3	29.6	1.3	2.2
100	9.6	1.3	21.4	1.3	2.5	10.1	1.2	21.2	1.3	2.3
250	8.5	1.2	12.6	1.3	2.0	6.7	1.1	11.1	0.9	1.6
500	5.0	1.3	7.0	1.3	1.7	5.9	1.3	8.2	1.4	1.6
1000	4.3	1.4	5.1	1.4	1.6	4.1	1.1	5.2	1.2	1.5
5000	2.1	1.4	2.3	1.4	1.3	1.6	1.1	1.8	1.1	1.0
	*ϕ* = 80					*ϕ* = 120				
	*α* = 10%									
50	31.6	10.9	48.9	10.4	8.0	31.7	9.6	50.1	9.2	7.7
100	27.7	10.3	38.9	10.3	7.9	28.1	10.4	38.1	10.3	7.5
250	22.8	10.8	27.3	10.8	8.3	22.1	10.5	26.1	10.7	8.9
500	18.7	10.1	20.8	10.0	9.5	18.5	10.2	20.6	10.3	9.4
1000	14.8	10.5	15.8	10.4	9.4	15.5	10.0	16.7	9.9	9.9
5000	11.3	9.7	11.7	9.7	9.9	12.6	10.8	12.8	10.8	10.3
	*α* = 5%									
50	22.3	5.3	41.6	5.6	5.4	22.1	4.9	42.1	4.5	5.2
100	20.5	5.2	31.5	5.1	4.8	20.3	5.3	31.0	5.4	4.8
250	16.0	5.3	20.7	5.1	4.8	15.2	5.3	20.0	5.2	5.1
500	12.4	5.1	14.8	4.9	4.8	12.1	5.0	14.4	5.1	5.2
1000	10.0	5.2	10.9	5.2	5.4	9.6	5.3	10.7	5.4	5.6
5000	6.2	5.0	6.5	5.0	5.2	7.1	5.5	7.4	5.6	5.4
	*α* = 1%									
50	10.7	1.1	30.4	1.1	2.9	9.6	0.9	29.7	1.0	2.7
100	9.7	1.2	20.8	0.9	2.2	10.0	1.2	21.0	1.2	2.1
250	7.7	0.9	12.1	1.0	1.8	7.7	1.1	12.0	1.1	1.9
500	5.3	1.1	7.0	1.2	1.6	5.4	1.2	7.5	1.2	1.9
1000	3.9	1.2	4.8	1.2	1.6	4.1	1.4	5.1	1.3	1.4
5000	2.1	1.4	2.2	1.3	1.2	2.1	1.2	2.3	1.2	1.1

The third and final set size simulations was performed using *μ* = 0.75 with the same precision values as before. We used *μ* = 0.75 (and not *μ* = 0.8) to avoid symmetry relative to the first scenario. The null rejection rates, expressed as percentages, are presented in Table 3. Overall, the results in this scenario are similar to those in Table 1 (*μ* = 0.2). The *ζ*_1_ and *ζ*_2_ tests are liberal when *n* ≤ 1000 and only become accurate with *n* = 5000. The *ζ*_1*B*_ and *ζ*_2*B*_ tests have the smallest size distortions. Such tests deliver accurate inferences even when *n* is small. For example, when *n* = 50, *ϕ* = 40 and *α* = 10%, their null rejection rates are 10.1% and 9.7%, respectively. It should also be noted that the *ζ*_3_ test exhibits conservative behavior when *n* ≤ 500. For example, with *n* = 500, *ϕ* = 20 and *α* = 10%, its null rejection rate is 8.7%.

**Table 3:** Null rejection rates (%), *μ* = 0.75.

*n*	*ζ*_1_	*ζ*_1*B*_	*ζ*_2_	*ζ*_2*B*_	*ζ*_3_	*ζ*_1_	*ζ*_1*B*_	*ζ*_2_	*ζ*_2*B*_	*ζ*_3_
	*ϕ* = 20					*ϕ* = 40				
	*α* = 10%									
50	21.6	10.8	52.3	10.3	5.6	18.0	10.1	49.3	9.7	5.7
100	20.7	9.6	37.1	9.8	5.9	20.0	10.4	37.7	10.1	6.0
250	18.8	10.1	26.0	9.9	7.2	18.3	10.5	27.1	10.5	7.2
500	16.5	9.4	20.3	9.4	8.7	16.1	9.9	20.6	9.5	7.9
1000	15.1	10.1	16.9	10.1	9.2	14.0	10.3	16.5	9.8	8.5
5000	11.2	9.7	11.5	9.7	10.0	12.2	10.8	12.7	11.0	9.3
	*α* = 5%									
50	12.9	5.4	44.5	5.4	3.5	10.5	5.5	42.3	4.9	3.4
100	13.2	4.9	30.4	4.7	3.6	12.1	5.2	30.8	5.4	3.3
250	12.9	5.0	20.0	5.0	4.4	12.3	5.0	20.8	5.4	4.1
500	10.5	4.7	14.2	4.9	4.6	10.4	4.9	14.6	4.5	4.1
1000	9.1	5.1	11.2	5.0	4.5	8.8	4.8	10.8	4.7	5.0
5000	6.3	4.8	6.6	4.9	4.8	6.5	5.3	7.1	5.2	5.1
	*α* = 1%									
50	4.8	1.3	31.8	1.4	1.8	4.4	1.3	29.3	1.1	1.4
100	4.5	0.9	20.0	0.8	1.4	4.2	1.3	20.2	1.1	1.2
250	5.5	1.2	11.8	1.2	1.6	4.5	0.9	12.2	1.3	1.4
500	4.2	1.0	7.1	1.0	1.7	3.9	1.1	7.1	1.2	1.5
1000	3.3	1.1	4.9	1.0	1.3	3.0	1.3	4.7	1.1	1.4
5000	1.8	1.0	2.0	0.9	1.2	1.8	1.1	2.1	1.2	1.2
	*ϕ* = 80					*ϕ* = 120				
	*α* = 10%									
50	15.5	10.4	48.9	10.5	4.0	14.4	10.8	49.7	10.5	4.6
100	16.4	10.3	38.0	9.9	5.2	14.5	10.3	37.9	10.2	5.3
250	15.3	9.8	26.7	10.0	5.6	14.5	10.4	27.4	10.9	5.6
500	14.6	10.1	20.0	10.4	7.2	13.3	10.5	19.8	10.5	6.7
1000	14.5	11.4	18.1	11.5	8.1	13.9	11.4	17.5	11.1	8.0
5000	12.0	10.7	12.9	10.7	10.3	11.3	10.2	12.3	10.3	9.5
	*α* = 5%									
50	9.5	5.4	42.6	5.3	2.5	8.7	5.5	42.9	5.3	2.8
100	9.4	5.3	31.5	5.4	3.0	8.3	5.3	30.4	5.1	2.9
250	9.2	5.0	19.9	5.3	3.3	8.7	5.6	20.6	5.4	3.1
500	9.1	5.3	14.8	5.5	4.0	8.3	5.4	14.7	5.9	3.4
1000	8.8	5.6	12.5	5.4	4.1	8.3	5.8	11.9	5.7	3.9
5000	6.8	5.6	7.4	5.5	5.0	5.9	5.2	6.7	5.1	4.7
	*α* = 1%									
50	3.9	1.3	29.7	1.2	0.9	3.2	1.1	31.2	1.0	0.9
100	3.3	1.0	20.6	1.4	1.2	2.8	1.1	20.0	0.9	1.0
250	3.6	1.4	11.4	1.3	1.3	3.0	1.3	12.5	1.3	0.9
500	3.3	1.2	7.5	1.1	1.2	3.1	1.3	8.0	1.2	0.8
1000	3.1	1.3	5.2	1.4	1.1	2.7	1.2	5.1	1.2	0.9
5000	1.7	1.1	2.0	1.2	1.3	1.6	1.2	2.1	1.2	1.1

The results presented above show that, in general, the *ζ*_1_ test exhibits less liberal behavior when the mean of the distribution is not in the middle of the standard unit interval. For instance, when *ϕ* = 120, *n* = 250 and *α* = 10%, the test’s null rejection rates for *μ* = 0.2, 0.5, 0.75 are 12.7%, 22.1% and 14.5%, respectively. Recall that the beta density is symmetric if *μ* = 0.5 and asymmetric otherwise. It seems that the *ζ*_1_ test incorrectly finds increasing evidence against the beta model as the distribution becomes more symmetric. The results also show that the *ζ*_2_ test is quite liberal in all scenarios, especially when the sample size is small. Finally, the *ζ*_3_ test becomes more conservative as the distribution mean moves away from 0.5. For example, when *ϕ* = 80, *n* = 500 and *α* = 10%, the test’s null rejection rates for *μ* = 0.2, 0.5, 0.75 are 7.0%, 9.5% and 7.2%, respectively.

**Fig 1:**
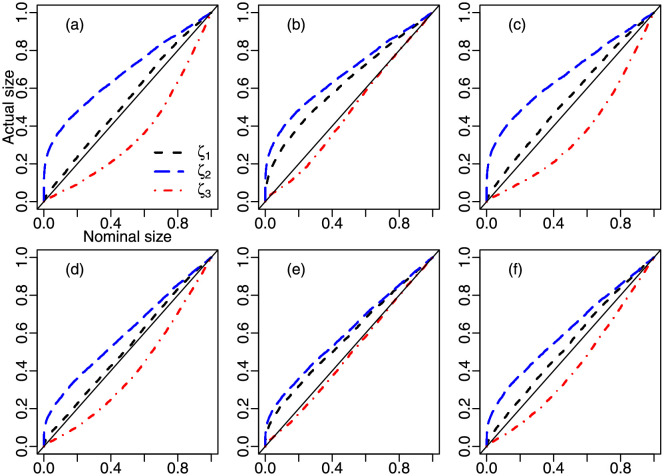
*P*-value plots; panel (a): ℬ(0.2,120) and *n* = 100, panel (b) ℬ(0.5,120) and *n* = 100, panel (c) ℬ(0.75,120) and *n* = 100, panel (d) ℬ(0.2,120) and *n* = 250, panel (e) ℬ(0.5,120) and *n* = 250, panel (f) ℬ(0.75,120) and *n* = 250.

Fig 1 contains *p*-value plots for the *ζ*_1_, *ζ*_2_ and *ζ*_3_ tests corresponding to different values of *μ*. The sample sizes are *n* = 100, 250 and *ϕ* = 120. The three curves move closer to the diagonal line when the sample increases from *n* = 100 to *n* = 250, thus indicating that the tests’ size distortions for all nominal sizes decrease as *n* increases. It is also clear that *ζ*_1_ and *ζ*_2_ are liberal and *ζ*_3_ is conservative regardless of the value of *α*, *ζ*_1_ being less size-distorted than *ζ*_2_, especially when the underlying beta law is asymmetric (*μ* ≠ 0.5). Interestingly, for all values of *α*, under distributional asymmetry (symmetry), *ζ*_1_ (*ζ*_3_) is the most accurate test.

We will now shift the focus to the tests’ powers, i.e., to their ability of correctly identifying that the null hypothesis is false. In these simulations, the true data-generating process is not the standard beta law, i.e., it is not the beta distribution with constant parameters. Since the *ζ*_1_ and *ζ*_2_ tests are oftentimes considerably size-distorted, they are carried out using exact (not asymptotic) critical values obtained from the size simulations. The significance levels are *α* = 10%, 5%.

At the outset, we use the Kumaraswamy law (see [7]), KW(ω,ϕ), as the true data-generating mechanism. Here, *ω* is the distribution median and *ϕ* is a precision parameter. The parameter values are (i) *ω* = 0.2 and *ϕ* = 5, 7.5, (ii) *ω* = 0.5 and *ϕ* = 10, 15, and (iii) *ω* = 0.75 and *ϕ* = 15, 25. The tests’ non-null rejection rates are presented in Table 4. All entries are percentages. The figures in this table show that the tests’ powers are similar for *n* ≥ 100, being close to 100% when *n* ≥ 250. When *n* = 50, the *ζ*_3_ test is generally the most powerful test. The reported results also show that the tests’ powers increase with *ϕ*. That is, higher precision translates into more powerful tests. Also, when *ω* = 0.2, the *ζ*_3_ test exhibits slightly higher powers than the *ζ*_1_ and *ζ*_1*B*_ tests, and these in turn exhibit noticeably higher powers than *ζ*_2_ and *ζ*_2*B*_. For illustration, with *ω* = 0.2, *ϕ* = 7.5, *n* = 100 and *α* = 5%, the non-null rejection rates of the *ζ*_1_, *ζ*_1*B*_, *ζ*_2_, *ζ*_2*B*_ and *ζ*_3_ tests are 61.3%, 65.2%, 56.7%, 56.8% and 69.7%, respectively. Here, *ζ*_3_ is the best performer. It is also noteworthy that *ζ*_3_ is the most powerful test when *ω* = 0.5 for all values of *ϕ* and *α*. Additionally, it is seen that the *ζ*_2_ and *ζ*_2*B*_ tests are more powerful than the *ζ*_1_ and *ζ*_1*B*_ tests. Finally, when *ω* = 0.75, for all values of *α* and *ϕ*, the *ζ*_1_, *ζ*_1*B*_ and *ζ*_3_ tests display similar powers, which are considerably higher than those of *ζ*_2_ and *ζ*_2*B*_.

**Table 4:** Non-null rejection rates (%), data generated from KW(ω,ϕ).

*n*	*ζ*_1_	*ζ*_1*B*_	*ζ*_2_	*ζ*_2*B*_	*ζ*_3_	*ζ*_1_	*ζ*_1*B*_	*ζ*_2_	*ζ*_2*B*_	*ζ*_3_
	*ω* = 0.2 and *φ* = 5					*ω* = 0.2 and *φ* = 7.5				
	*α* = 10%									
50	37.6	36.6	29.8	30.2	37.6	48.9	52.7	41.5	42.3	49.0
100	59.4	57.7	56.4	56.3	65.3	76.6	80.2	72.0	72.6	79.0
250	93.1	92.7	96.2	95.9	98.0	98.4	98.9	99.3	99.2	99.4
500	99.5	99.4	100.0	100.0	100.0	99.9	99.9	100.0	100.0	100.0
1000	100.0	100.0	100.0	100.0	100.0	100.0	100.0	100.0	100.0	100.0
5000	100.0	100.0	100.0	100.0	100.0	100.0	100.0	100.0	100.0	100.0
	*α* = 5%									
50	23.9	21.4	17.6	17.3	29.6	35.1	36.9	28.0	28.1	40.8
100	40.3	39.0	38.8	39.0	54.6	61.3	65.2	56.7	56.8	69.7
250	82.8	81.8	91.6	90.5	95.6	94.6	96.5	97.9	97.5	98.8
500	97.8	97.7	99.8	99.8	100.0	99.4	99.7	100.0	100.0	100.0
1000	100.0	100.0	100.0	100.0	100.0	99.9	100.0	100.0	100.0	100.0
5000	100.0	100.0	100.0	100.0	100.0	100.0	100.0	100.0	100.0	100.0
	*ω* = 0.5 and *ϕ* = 10					*ω* = 0.5 and *ϕ* = 15				
	*α* = 10%									
50	20.5	22.2	32.8	33.7	40.9	29.1	27.8	46.2	44.0	51.9
100	43.3	45.2	59.2	60.1	69.1	54.2	55.3	73.8	74.0	80.9
250	92.8	93.7	96.2	96.3	98.1	96.9	97.1	99.3	99.3	99.7
500	100.0	100.0	100.0	100.0	100.0	100.0	100.0	100.0	100.0	100.0
1000	100.0	100.0	100.0	100.0	100.0	100.0	100.0	100.0	100.0	100.0
5000	100.0	100.0	100.0	100.0	100.0	100.0	100.0	100.0	100.0	100.0
	*α* = 5%									
50	8.9	10.2	20.0	21.3	32.6	12.5	12.4	31.4	30.3	42.9
100	24.0	25.6	43.8	44.4	59.0	33.7	34.6	58.5	59.7	71.9
250	83.0	83.4	91.5	91.4	95.7	91.3	91.2	97.6	97.6	98.9
500	99.8	99.8	99.9	99.9	99.9	99.9	99.9	100.0	100.0	100.0
1000	100.0	100.0	100.0	100.0	100.0	100.0	100.0	100.0	100.0	100.0
5000	100.0	100.0	100.0	100.0	100.0	100.0	100.0	100.0	100.0	100.0
	*ω* = 0.75 and *ϕ* = 15					*ω* = 0.75 and *ϕ* = 25				
	*α* = 10%									
50	32.7	30.7	23.1	24.0	25.7	49.1	51.9	37.6	38.2	41.9
100	55.8	52.4	39.7	39.3	47.7	77.9	78.6	64.1	64.0	69.2
250	88.9	87.8	79.7	79.0	86.5	99.2	99.2	97.3	97.7	98.3
500	99.2	99.1	98.6	98.6	99.1	100.0	100.0	100.0	100.0	100.0
1000	100.0	100.0	100.0	100.0	100.0	100.0	100.0	100.0	100.0	100.0
5000	100.0	100.0	100.0	100.0	100.0	100.0	100.0	100.0	100.0	100.0
	*α* = 5%									
50	18.2	16.9	13.9	14.5	19.3	32.3	34.4	25.3	25.5	32.3
100	39.1	36.4	25.4	27.1	38.6	64.3	65.3	49.1	49.1	60.3
250	81.6	78.2	64.3	65.7	79.3	97.7	98.2	93.4	93.5	96.5
500	98.0	97.7	95.6	95.6	98.1	100.0	100.0	99.9	99.9	100.0
1000	100.0	100.0	100.0	100.0	100.0	100.0	100.0	100.0	100.0	100.0
5000	100.0	100.0	100.0	100.0	100.0	100.0	100.0	100.0	100.0	100.0

**Fig 2:**
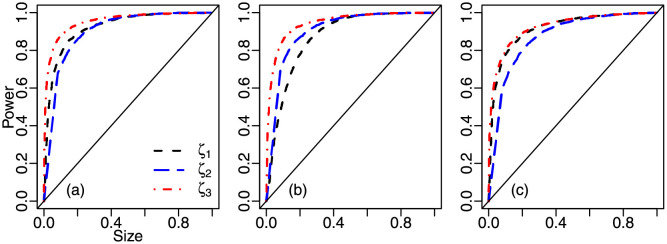
Size-power plots, KW(ω,ϕ), *n* = 100; panel (a): KW(0.2,7.5), panel (b): KW(0.5,15), panel (c): KW(0.75,25).

Fig 2 contains size-power plots for *ζ*_1_, *ζ*_2_ and *ζ*_3_. The sample size is *n* = 100 and the empirical powers were computed using KW(ω,ϕ) data-generating processes. The tests’ powers are very similar for empirical sizes in excess of 0.4. For empirical sizes up to 0.4, *ζ*_3_ is the clear winner, especially in the left and middle panels; in the right panel, the curves relative to *ζ*_1_ and *ζ*_3_ nearly coincide, both clearly lying above that of *ζ*_2_. Also, *ζ*_1_ is the worst performer when the distribution median lies at the center of the standard unit interval, i.e., *ω* = 0.5; see panel (b).

In the second scenario of power simulations, all samples are randomly generated from the unit Weibull law (see [9]), UW(ω,ϕ), where *ω* is the distribution median and *ϕ* is a precision parameter. For brevity, we only report results obtained using *ω* = 0.2, 0.5, 0.75 and *ϕ* = 5. The tests’ non-null rejection rates are given in Table 5. All entries are percentages. It is worth noticing that all empirical powers are nearly equal to 100% when *n* = 250. When *ω* = 0.2, *ζ*_1_ is the best performer. The *ζ*_3_ test is slightly more powerful than the other tests when *ω* = 0.5 and 0.75.

**Table 5:** Non-null rejection rates (%), data generated from UW(ω,ϕ).

*n*	*ζ*_1_	*ζ*_1*B*_	*ζ*_2_	*ζ*_2*B*_	*ζ*_3_	*ζ*_1_	*ζ*_1*B*_	*ζ*_2_	*ζ*_2*B*_	*ζ*_3_	*ζ*_1_	*ζ*_1*B*_	*ζ*_2_	*ζ*_2*B*_	*ζ*_3_
	*ω* = 0.2 and *ϕ* = 5					*ω* = 0.5 and *ϕ* = 5					*ω* = 0.75 and *ϕ* = 5				
	*α* = 10%														
50	68.0	60.9	54.1	53.3	53.4	24.1	26.2	41.2	42.4	49.6	45.6	44.0	39.7	40.2	47.1
100	94.8	91.1	83.9	83.6	85.1	55.6	57.4	74.7	75.2	81.2	72.2	68.9	72.0	71.3	77.6
250	100.0	100.0	99.9	99.9	99.9	97.4	97.9	99.5	99.6	99.8	96.9	95.8	99.3	99.4	99.5
500	100.0	100.0	100.0	100.0	100.0	100.0	100.0	100.0	100.0	100.0	99.7	99.4	100.0	100.0	100.0
1000	100.0	100.0	100.0	100.0	100.0	100.0	100.0	100.0	100.0	100.0	100.0	100.0	100.0	100.0	100.0
5000	100.0	100.0	100.0	100.0	100.0	100.0	100.0	100.0	100.0	100.0	100.0	100.0	100.0	100.0	100.0
	*α* = 5%														
50	51.7	43.3	40.7	40.4	44.0	10.2	11.8	27.5	28.5	40.3	28.2	26.0	25.8	25.9	37.9
100	89.4	82.7	72.3	72.9	77.4	34.8	36.3	60.9	61.1	72.9	54.8	49.3	56.3	55.7	69.0
250	100.0	99.9	99.5	99.5	99.7	92.8	92.7	98.4	98.4	99.1	91.0	89.1	97.9	97.8	98.8
500	100.0	100.0	100.0	100.0	100.0	100.0	100.0	100.0	100.0	100.0	98.5	98.3	100.0	100.0	100.0
1000	100.0	100.0	100.0	100.0	100.0	100.0	100.0	100.0	100.0	100.0	99.9	99.9	100.0	100.0	100.0
5000	100.0	100.0	100.0	100.0	100.0	100.0	100.0	100.0	100.0	100.0	100.0	100.0	100.0	100.0	100.0

**Fig 3:**
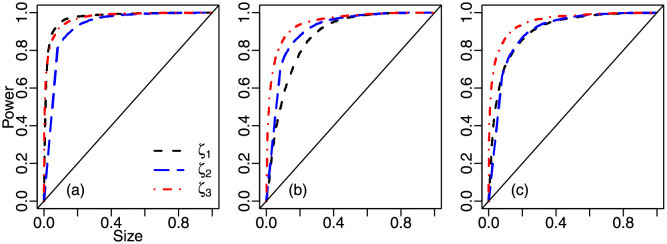
Size-power plots, UW(ω,ϕ), *n* = 100; panel (a): UW(0.2,5), panel (b): UW(0.5,5), panel (c): UW(0.75,5).

Size-power plots are presented in Fig 3. The sample size is *n* = 100 and the tests’ empirical powers were computed using unit Weibull data-generating mechanisms. In Fig 3 panel (a), the size-power curves of the *ζ*_1_ and *ζ*_3_ tests are clearly above that of the *ζ*_2_ test for empirical sizes up to approximately 40%. In panel (b) of Fig 3, for empirical sizes up to about 50%, the curve of the *ζ*_3_ test is above the curve of the *ζ*_2_ test, which in turn is above that of the *ζ*_1_ test. Finally, panel (c) of Fig 3 clearly favors *ζ*_3_ for empirical sizes up to approximately 50%.

The next set of power simulation results was obtained using simplex (see [8]) data-generating mechanisms: all samples are randomly generated from S(μ,σ), where *μ* is the distribution mean and *σ* is the dispersion parameter. For brevity, we only present results for *μ* = 0.75 and *σ* = 2. The tests’ non-null rejection rates, expressed as percentages, can be found in Table 6. It is noteworthy that the powers of the *ζ*_1_, *ζ*_1*B*_, *ζ*_2_ and *ζ*_2*B*_ tests are quite high for *n* ≥ 250. Also, the *ζ*_3_ test is clearly less powerful than the competing tests. For example, when *n* = 100 and *α* = 10%, the powers of the *ζ*_1_, *ζ*_1*B*_, *ζ*_2_ and *ζ*_2*B*_ tests exceed 60% whereas that of the *ζ*_3_ test is approximately equal to 22%.

**Table 6:** Non-null rejection rates (%), data generated from S(μ,σ).

*n*	*ζ*_1_	*ζ*_1*B*_	*ζ*_2_	*ζ*_2*B*_	*ζ*_3_
	*μ* = 0.75 and *σ* = 2				
	*α* = 10%				
50	39.5	27.8	42.0	40.4	9.8
100	80.6	62.9	68.0	66.2	22.2
250	99.3	98.1	97.5	97.6	79.0
500	100.0	100.0	100.0	100.0	99.6
1000	100.0	100.0	100.0	100.0	100.0
5000	100.0	100.0	100.0	100.0	100.0
	*α* = 5%				
50	20.4	13.7	28.9	26.5	5.9
100	61.6	37.0	53.7	51.3	12.6
250	97.6	94.7	94.8	94.0	58.0
500	100.0	100.0	100.0	100.0	98.8
1000	100.0	100.0	100.0	100.0	100.0
5000	100.0	100.0	100.0	100.0	100.0

**Fig 4:**
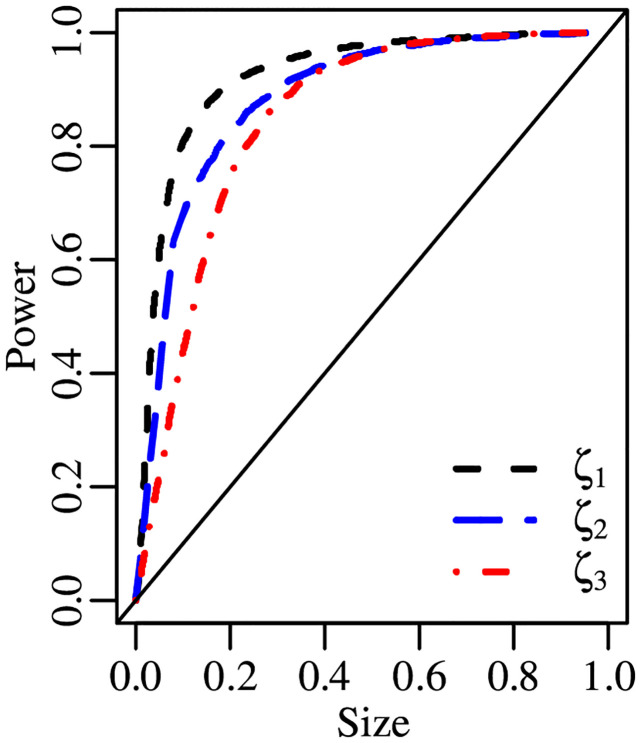
Size-power plot, S(μ,σ), *n* = 100.

We present size-power plots constructed using the tests’ empirical powers under simplex laws in Fig 4. The sample size is *n* = 100. It can be seen that the curves relative to the *ζ*_1_ and *ζ*_2_ tests are similar. They both lie considerably above that of the *ζ*_3_ test for effective sizes up to 40%.

Next, we consider the case in which the data are generated from the beta law but with a regression structure for the mean. That is, we use the beta regression model introduced by [18] as the true model. Here, log(*μ*_*t*_/(1− *μ*_*t*_)) = *β*_1_ + *β*_2_*x*_*t*2_. The true parameter values are *β*_1_ = −0.25, *β*_2_ = 0.5 and *ϕ* = 120. The covariate values were generated from *LN*(0, 0.5), i.e., as realizations from the log-normal distribution with parameters 0 and 0.5. Table 7 contains the tests’ non-null rejection rates, all expressed as percentages. In general, all tests have high powers when the sample size is not very small. In particular, for *n* = 250 and *α* = 10%, the tests have powers close to or equal to 100%. When *n* = 50, *ζ*_1_ is clearly less powerful than *ζ*_2_ and *ζ*_3_.

**Table 7:** Non-null rejection rates (%), data generated from the beta distribution with a mean regression structure.

*n*	*ζ*_1_	*ζ*_1*B*_	*ζ*_2_	*ζ*_2*B*_	*ζ*_3_
	*α* = 10%				
50	37.3	42.1	56.1	53.3	47.8
100	89.3	90.9	91.0	90.7	91.8
250	97.7	97.7	100.0	100.0	100.0
500	100.0	100.0	100.0	100.0	100.0
1000	100.0	100.0	100.0	100.0	100.0
5000	100.0	100.0	100.0	100.0	100.0
	*α* = 5%				
50	16.3	19.9	40.6	38.2	30.2
100	72.1	75.0	81.8	82.3	79.6
250	87.3	87.7	99.6	99.5	100.0
500	99.9	99.9	100.0	100.0	100.0
1000	100.0	100.0	100.0	100.0	100.0
5000	100.0	100.0	100.0	100.0	100.0

**Fig 5:**
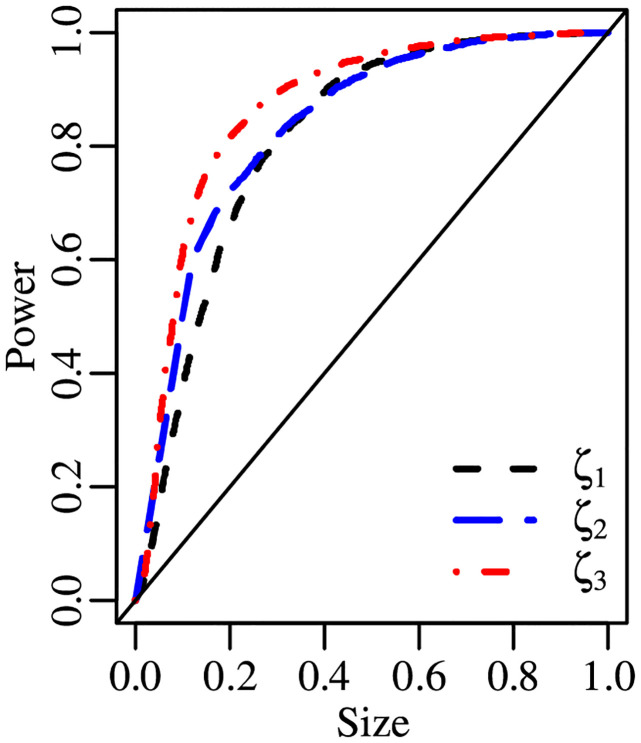
Size-power plot, $ℬ(\mu_{t},\phi )$, *n* = 50.

In Fig 5, we present the size-power plot of *ζ*_1_, *ζ*_2_ and *ζ*_3_ for *n* = 50. In general, the tests have similar powers when the effective size is smaller than 20% or larger than 60%. In the middle region of the graph, *ζ*_3_ is the most powerful test.

We also performed simulations using the inflated beta distribution introduced by [25] as the true model. It combines continuous and discrete components, and is used when *Y* assumes values in [0, 1), (0, 1] or [0, 1] (inflation at zero, inflation at one, and double inflation, respectively). A common practice is to fit the standard beta distribution after replacing the inflated data points by [*Y*_*t*_(*n*−1) + 0.5]/*n*; see [26]. We consider inflation at zero with Pr(*Y* = 0) = λ. After the data were generated, all inflated values (zeros) were replaced by 0.5/*n*, and then the standard beta law was fitted. The null hypothesis is false since the beta model is not the true data generating process. We wish to evaluate the information matrix tests’ ability to detect that the beta model is misspecified. Data generation was carried out using *μ* = 0.5, *ϕ* = 20 and λ = 0.025. We will not present the simulation results for brevity, but we note that the information matrix tests proved to be very powerful in this setting with non-null rejection rates close to 100% at *α* = 5% for *n* = 100.

Overall, the results presented above favor the *ζ*_1*B*_, *ζ*_2*B*_ and *ζ*_3_ tests. The *ζ*_1_ and *ζ*_2_ tests typically display very large size distortions and their use should be avoided except when *n* is large. Regarding the *ζ*_1*B*_, *ζ*_2*B*_ and *ζ*_3_ tests, we note that the latter may be considerably conservative for some beta law parameter values. As a result, we recommend the use of the *ζ*_1*B*_ and *ζ*_2*B*_ tests in empirical analyses. Such tests showed good control of the type I error frequency and also good power in situations in which the data-generating process is not beta, in particular when *n* ≥ 250.

It is also possible to test the null hypothesis that the variable of interest is beta-distributed using two alternative tests, namely: Anderson-Darling (AD) and Cramér-von Mises (CVM). They are usually carried with the modification proposed by [27], which accounts for unknown parameters in the distribution under test (in our case, beta). We performed Monte Carlo simulations to assess the finite sample behaviors of such tests using the configurations previously described. We do not present such results for brevity. We note, however, that both tests are conservative, i.e., their null rejection rates are smaller than the significance levels. For instance, when *n* = 100 (*n* = 500) the AD and CVM null rejection rates at the 10% significance level are, respectively, 7.6% and 6.3% (9.2% and 8.5%). Also, such non-parametric tests are substantially less powerful than the information matrix tests introduced in this paper. For instance, when the true data-generating process is UW(0.5,7.5) (KW(0.5,15)), the AD and CVM non-null rejection rates at *α* = 10% are, respectively, 29.1% and 42.0% (28.9% and 39.3%) when *n* = 5000.

## Covid-19 mortality rates in the US

We will now present and discuss an analysis of Covid-19 mortality rates in the US. We use the three information matrix tests to determine whether the standard beta model provides an adequate representation of the data. We will also briefly comment on inferences drawn from the AD and CVM tests. Maximization of the beta log-likelihood function was performed using the BFGS method with analytical first derivatives. We used *B* = 1000 bootstrap replications for performing the *ζ*_1*B*_, *ζ*_2*B*_ and *ζ*_3_ information matrix tests. In what follows, we model state and county level data for three time periods. In each case, we will report the information matrix tests’ *p*-values, the point estimates of the beta parameters and their standard errors. We report clustered standard errors computed using information on each state’s region and on to each county’s state.

The Covid-19 epidemic began in late 2019. It is estimated that approximately 247 million people had been infected with the new coronavirus by October 2021. The United States was the first country in the Americas to face a serious public health crisis brought on by the new coronavirus. In December 2020, on the 14th to be exact, the US government began a campaign to vaccinate healthcare workers and followed by vaccinating the general population. Covid-19 death rates started to decrease as vaccination progressed.

Our variable of interest are Covid-19 mortality rates per one hundred people. At the outset, we will work with statewide data, i.e., we use data on the 50 US states (*n* = 50). The death rates were computed using the cumulative number of deaths between January 22 and December 14 of 2020. We refer to this period as ‘period 1’. The source of the data on Covid-19 deaths is the Centers for Disease Control and Prevention (https://data.cdc.gov/). Data on state populations in 2020 were obtained from [10]. Since the sample size is small, we only consider bootstrap-based information matrix testing inferences. We wish to determine whether the univariate beta model provides an adequate representation of the data. The model has a simple structure and is based on the assumption that the observations are i.i.d. Can it provide an acceptable and useful representation of the US Covid-19 mortality rates?

**Fig 6:**
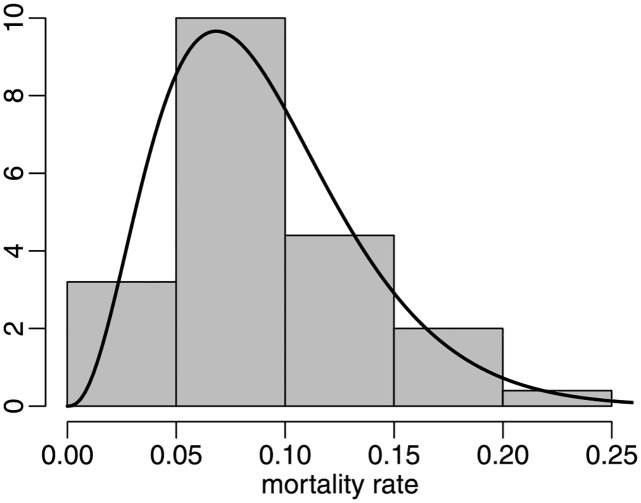
Histogram and fitted beta density, period 1, state data.

The minimum, mean, median, and maximum mortality rates, and the standard deviation are 0.0164, 0.0903, 0.0894, 0.2001 and 0.0423, respectively. The maximal value corresponds to New Jersey. The maximum likelihood estimates of the beta parameters (clustered standard errors in parentheses) are $\hat{\mu }=0.0900$ (0.0099) and $\hat{\phi }=39.8208$ (15.5240). The *p*-values of the *ζ*_1*B*_, *ζ*_2*B*_ and *ζ*_3_ tests of correct beta specification are 0.2870, 0.6070 and 0.4472, respectively. The model is not rejected at the usual significance levels. We thus conclude that it adequately represents the US state mortality rates. In Fig 6 we present the histogram of the mortality rates together with the beta density evaluated at the maximum likelihood estimates. The estimated density clearly provides a good approximation to the data histogram.

**Fig 7:**
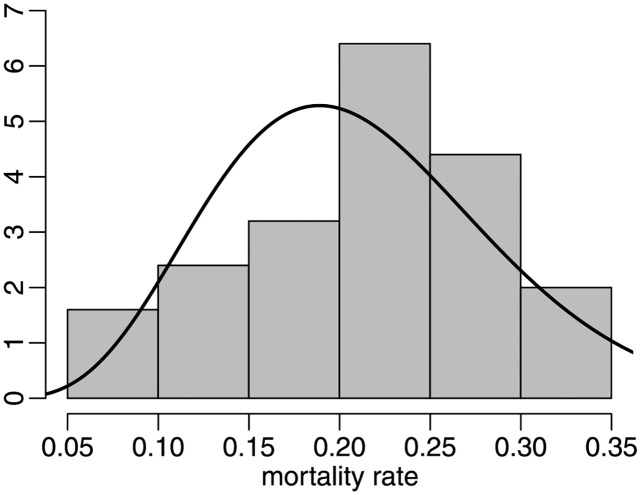
Histogram and fitted beta density, period 2, state data.

The previous analysis was performed using mortality rates computed up to December 14, 2020. Next, we will conduct a similar analysis, but based on more recent data. We consider state mortality rates calculated using data from January 22, 2020 to October 31, 2021. We refer to this more extended time period as ‘period 2’. The minimum, mean, median, maximum and standard deviation values are 0.0550, 0.2120, 0.2227, 0.3370 and 0.0709, respectively. The maximum likelihood point estimates are $\hat{\mu }=0.2112$ (0.0199) and $\hat{\phi }=27.8235$ (8.9936). The estimated precision is now approximately 30% smaller than in the previous scenario. The *p*-values of the *ζ*_1*B*_, *ζ*_2*B*_ and *ζ*_3_ tests are 0.0600, 0.0570 and 0.0269, respectively. All tests reject the correct specification of the univariate beta model at the 10% significance level; *ζ*_3_ rejects $H_{0}$ at 5%. Fig 7 presents the data histogram and the estimated beta density. The estimated beta density does not adequately represent the data asymmetry.

Unlike the previous results, all tests now reject the beta distribution at *α* = 10%. The data now cover two very different periods, namely: before and after the start of the nationwide vaccination campaign. There is thus clear data heterogeneity. The much smaller estimated precision (approx. 28 vs approx. 40) is probably due to such heterogeneity.

The mortality rates in the two periods show high positive correlation (0.8252), as expected, given the cumulative nature of the observations. The univariate beta model is not rejected by the information matrix tests when the shorter time period is used. It thus provides a good description of the statewide Covid-19 mortality rates. The second time period, however, covers the Covid-19 vaccination campaign. Since the reach and impact of such a campaign was uneven across the 50 states, for reasons that include partisan political connotations and other factors, Covid-19 mortality rates greatly differ before and after the beginning of the immunization campaign. There is thus clear heterogeneity in the two periods.

**Fig 8:**
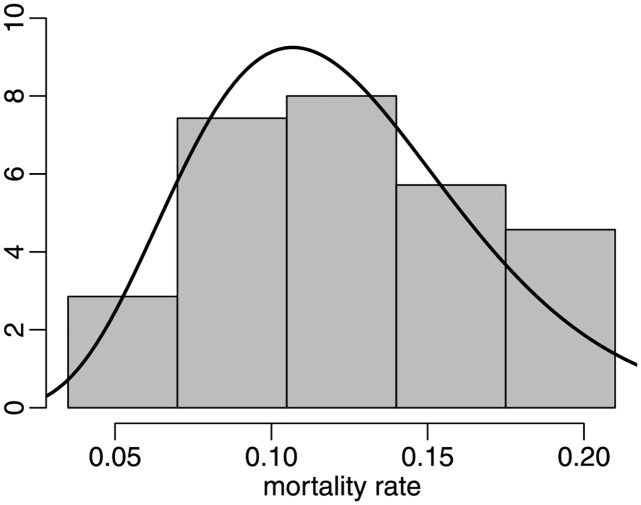
Histogram and fitted beta density, period 3.

The two analyses presented so far are based on cumulative time periods, namely: (i) January 22 to December 14, 2020 (without vaccination) and (ii) January 22, 2020 to October 31, 2021 (without and with vaccination). In the following, we will only consider the most recent period (December 15, 2020 to October 31, 2021), ‘period 3’. The minimal and maximal values are 0.0385 and 0.1985 whereas the mean and median values are 0.1218 and 0.1174, respectively; the standard deviation is 0.0432. The maximum likelihood estimates of *μ* and *ϕ* are 0.1216 (0.0153) and 52.8670 (11.1468), respectively. The estimated precision is even larger than that obtained by only considering the pre-vaccination time period (approx. 53 vs approx. 40). Recall that much lower precision was obtained when the longest time period was considered (approx. 28). The *ζ*_1*B*_, *ζ*_2*B*_ and *ζ*_3_ *p*-values are, respectively, 0.5900, 0.2860 and 0.5087. These large *p*-values indicate that there is very little evidence against the beta law. We thus conclude that despite the impact of vaccination on Covid-19 mortality, the univariate beta model still provides a good representation of the data. The data histogram and the fitted beta density are presented in Fig 8. Visual inspection of such a figure suggests that the beta law yields a reasonably good data fit. Interestingly, there is less skewness than in the previous two cases.

**Fig 9:**
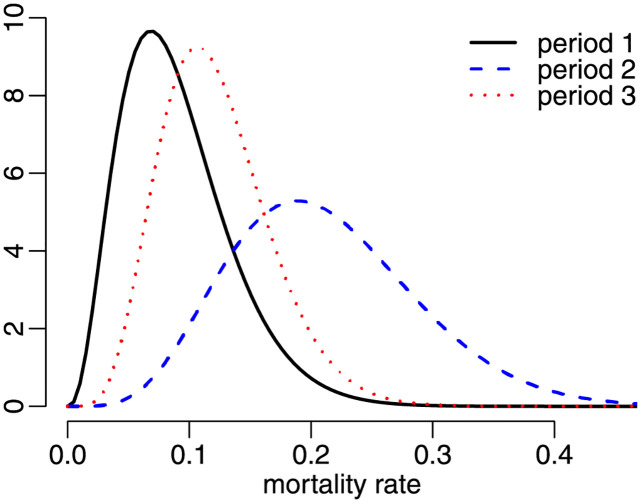
Fitted beta densities, state data.

The three fitted beta densities are presented in Fig 9. Notice that the estimated densities for periods 1 and 3 are similarly shaped and with somewhat similar precisions. By contrast, the fitted beta density obtained using data that cover both the period in which there was no vaccination and that of the vaccination drive is much more disperse. As noted earlier, heterogeneity in the data leads to poor data fit. The information matrix tests indicated that the beta model yields an adequate data representation in periods 1 and 3, but in for period 2. It seems that the tests correctly detected that the heterogeneous nature of the data renders the beta law unable to adequately represent Covid-19 mortality rates.

We presented above an analysis of statewide Covid-19 mortality data in the US. The inferences obtained from the information matrix tests were quite informative. Such tests indicated that the beta law is able to adequately represent the data in two disjoint periods—before and after the start of the nationwide vaccination campaign —, but not when the two periods are combined.

In what follows we will use death rates per 100 persons computed for US counties for periods 1, 2 and 3. The data on the cumulative total of deaths was obtained from the New York Times repository (https://github.com/nytimes/covid-19-data). In order to avoid inaccurate records, we only considered, in each time period, counties with at least one Covid-19 death and at least 15000 inhabitants. The sample sizes for periods 1, 2 and 3 are *n* = 2073, *n* = 2080 and *n* = 2080 respectively. Since the sample sizes are large, we will use all tests, i.e., *ζ*_1_, *ζ*_1*B*_, *ζ*_2_, *ζ*_2*B*_ and *ζ*_3_. Mortality rates were calculated using the estimated populations in 2020 obtained from the Economic Research Service of the US Department of Agriculture (https://www.ers.usda.gov).

Initially, we will consider period 1. The minimum, mean, median, maximum and standard deviation values of the mortality rates are 0.0013, 0.0883, 0.0764, 0.4554 and 0.0596, respectively. The maximum likelihood estimates are $\hat{\mu }=0.0884$ (0.0055) and $\hat{\phi }=22.1618$ (1.9529). The *ζ*_1_, *ζ*_1*B*_, *ζ*_2_, *ζ*_2*B*_ and *ζ*_3_ tests’ *p*-values are 0.0978, 0.1370, 0.0927, 0.1540 and 0.1022, respectively. No test rejects the beta law at *α* = 5%. The tests that use bootstrap resampling also do not reject such a hypothesis at *α* = 10%. The *p*-values of the tests that use asymptotic critical values are slightly smaller than 0.10. Overall, we conclude that Covid-19 mortality rates can be adequately represented by the beta law in period 1.

We will now consider the second period. The minimum, mean, median, maximum and standard deviation values are, respectively, 0.0122, 0.2513, 0.2418, 0.7376 and 0.1133. Also, $\hat{\mu }=0.2512$ (0.0118) and $\hat{\phi }=13.3931$ (1.1877). The estimate of *ϕ* is approximately 40% smaller than in the previous scenario. There is thus considerably more uncertainty. The *p*-values of the *ζ*_1_, *ζ*_1*B*_, *ζ*_2_, *ζ*_2*B*_ and *ζ*_3_ tests are 0.0157, 0.0570, 0.0155, 0.0650 and 0.0087, respectively. The beta law is rejected at *α* = 1% (*α* = 5%) [*α* = 10%] by *ζ*_3_ (*ζ*_1_ and *ζ*_2_) [*ζ*_1*B*_ and *η*_2*B*_]. Overall, we conclude that the beta law does not provide an adequate data representation in period 2.

Next, we will perform inferences with data from period 3. The minimum, mean, median, maximum and standard deviation of the mortality rates are 0.0085, 0.1632, 0.1528, 0.4734 and 0.0786 respectively. The point estimates are $\hat{\mu }=0.1631$ (0.0083) and $\hat{\phi }=20.4761$ (1.5648). The *p*-values of the *ζ*_1_, *ζ*_1*B*_, *ζ*_2_, *ζ*_2*B*_, and *ζ*_3_ tests are 0.0529, 0.0830, 0.0360, 0.0750, and 0.0903, respectively. Except for *ζ*_2_, no test rejects the beta law at *α* = 5%. We thus conclude that it can be used to adequately represent county-level Covid-19 mortality rates in the third and final period. We will return to these results later.

**Fig 10:**
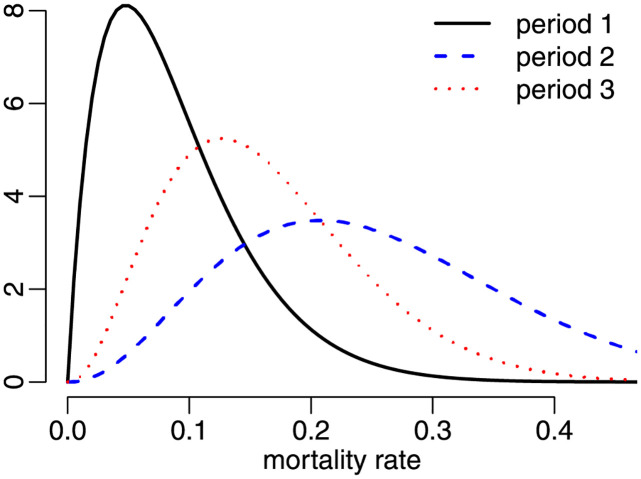
Fitted beta densities, county data.

Fig 10 contains the estimated densities for the three time periods obtained using county data. They are similar to those obtained using statewide data; see Fig 9. Notice that there is considerably more uncertainty when data from period 2 are used.

Interestingly, similar testing inferences were obtained with state and county data, namely: (i) the univariate beta model provides an adequate description of Covid-19 mortality rates with data either from prior to the nationwide vaccination drive or from when such a drive was under way; (ii) there is evidence against the correct specification of the beta model when Covid-19 mortality rates are computed using data that cover both periods (no vaccination and nationwide vaccination). The tests thus indicate that the beta distribution is not an adequate model for Covid-19 mortality rates under substantial data heterogeneity.

As noted earlier, we also performed the AD and CVM tests using both state and county data. The corresponding *p*-values for state data are: 0.4691 and 0.8734, period 1; 0.2277 and 0.4339, period 2; 0.9413 and 0.3360, period 3. With county data, we obtained the following *p*-values: 0.7414 and 0.5299, period 1; 0.3250 and 0.4856, period 2; 0.8765 and 0.8010, period 3. All *p*-values are quite large, and hence the beta model is accepted in all scenarios, i.e., for the three time periods and when state or county data are used. In particular, unlike the information matrix tests, the two non-parametric tests are not able to reject the beta model when there is marked data heterogeneity (period 2). By contrast, our tests indicate that the univariate beta model is only appropriate when there is reasonable homogeneity in the data (periods 1 and 3).

We will now further examine (i) the data heterogeneity that caused the rejection of beta law in period 2 and (ii) the acceptance of the beta law in period 3 when the vaccination drive was under way. As noted earlier, the Covid-19 mortality rates computed for period 2 cover two quite distinct periods: January 22, 2020 through December 14, 2020 (period 1) and December 15, 2020 through October 21, 2021 (period 3). (Recall that period 2 consists of the merging of periods 1 and 3.) The correlation coefficient between statewide death rates in periods 1 and 3 is weak: 0.3735. This small correlation strength is indicative that the mortality rates in such periods obey different dynamics. This was expected because, unlike what took place in period 3, there was no nationwide vaccination drive in period 1. Additionally, the states with the lowest mortality rates in period 1 (period 3) are Vermont, Hawaii, Maine, Oregon, and Utah (Vermont, Hawaii, New York, Alaska, and Maine) whereas those with the highest death rates in period 1 (period 3) are New Jersey, Massachusetts, Mississippi, Rhode Island, and North Dakota (Arizona, Alabama, West Virginia, Florida, and Georgia). Consider, e.g., New Jersey and Massachusetts. They are the states with the highest Covid-19 mortality rates in period 1, and yet their corresponding ranks in period 3 are 28 and 32. Arizona and Alabama display the highest death rates in period 3, and yet their ranks in period 1 are 14 and 9, respectively. Again, it is clear that the death rates in periods 1 and 3 (which are combined in period 2) are considerably heterogeneous.

Next, we will examine again Covid-19 mortality rates in period 3; in particular, we will examine the finding that the univariate beta model yields an adequate representation for such rates. There was a nationwide vaccination drive under way in period 3, and its reach negatively impacted death rates. We obtained data on the total number of fully vaccinated people by October 31, 2021. The source of the data is the Our World in Data repository (https://ourworldindata.org/us-states-vaccinations). The correlation between death and vaccination rates in period 3 is −0.5858 (state data). A natural question is: Given that mortality rates are negatively impacted by vaccination rates, why was the univariate beta model found to be correctly specified? Why use a fixed mean model if the distribution mean appears to be impacted by an explanatory variable (vaccination rate)? At the outset, we note that some states considerably weaken the inverse relationship between the two variables in period 3, namely: Alaska, Arizona, Florida, Massachusetts, North Dakota, and Rhode Island. In particular, the Arizona, Florida, Massachusetts, and Rhode Island (Alaska and North Dakota) Covid-19 mortality rates are higher (lower) than expected based on the corresponding vaccination levels. The inverse correlation between death and vaccination rates becomes considerably stronger when computed without such states: −0.7592 (state data). We removed from the data all counties of the six states that weaken the impact of vaccination reach on death rates, and performed the tests again. The *ζ*_1_, *ζ*_1*B*_, *ζ*_2_, *ζ*_2*B*_, and *ζ*_3_ *p*-values become 0.0289, 0.0600, 0.0162, 0.0530, and 0.0460, respectively. The *ζ*_1_, *ζ*_2_ and *ζ*_3_ tests now reject the univariate beta model at *α* = 5% whereas the *ζ*_1*B*_ and *ζ*_2*B*_*p*-values are only marginally larger than 0.05. Hence, there is now evidence against the model. Overall, the information matrix tests’ inferences suggest that, as long as the negative impact of vaccination reach on death rates is moderate (complete data), the beta law can be adequately used to represent Covid-19 mortality rates. When such a negative impact becomes more pronounced (incomplete data, counties of six states removed from the data), the univariate beta model no longer should be used. In that case, practitioners should search for a more elaborate model. By contrast, the two non-parametric tests continue to accept the univariate beta model even when the Alaska, Arizona, Florida, Massachusetts, North Dakota, and Rhode Island counties are not considered; the AD and CVM *p*-values are 0.3025 and 0.5788, respectively.

**Fig 11:**
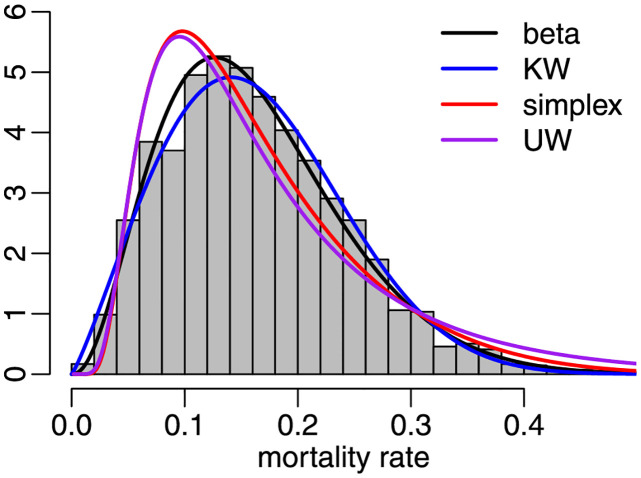
Histogram and fitted densities, period 3.

**Fig 12:**
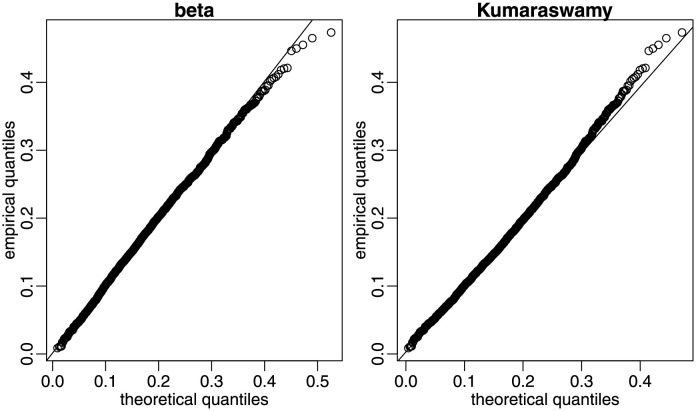
Quantile-quantile plots, period 3.

Finally, using the three county data samples, we compare the data fits yielded by the beta distribution to those obtained with the following alternative laws: Kumaraswamy, simplex, and unit Weibull. To that end, we computed, for each sample period and for each distribution, the values of the following information criteria: Akaike Information Criterion (AIC), Corrected Akaike Information Criterion (AICc), Bayesian Information Criterion (BIC), Hannan-Quinn Information Criterion (HQIC), Weighted-Average Information Criterion (WIC) and Empirical Information Criterion (EIC). The latter employs bootstrap resampling and proved to be very effective in dynamic beta modeling; see [28]. We used 1000 bootstrap replications, i.e., 1000 pseudo-samples were generated for computing the EIC values. We also computed the AD and CVM statistics. For all measures, smaller values indicate better data fits. The results are presented in Table 8. They show that, according to all information criteria (AIC, AICc, BIC, HQIC, WIC, and EIC), the best data fits in the three sample periods are yielded by the beta law. Considering the two non-parametric test statistics, in period 1 (period 2) [period 3], the beta model was the winner according to both of them (the runner-up according to both statistics, slightly behind the Kumaraswamy law) [the winner according to CVM and the runner-up according to AD, behind the Kumaraswamy model]. Considering the eight measures and the three sample periods, the beta law was the winner in 21 out of the 24 cases. Fig 11 contains the data histogram and the estimated beta density for period 3, as in Fig 8, together with the fitted Kumaraswamy (KW), simplex and unit Weibull (UW) densities. Visual inspection of the figure shows that the beta law best fits the data histogram. In order to further examine the two best data fits, we produced quantile-quantile (QQ) plots for the beta and Kumaraswamy laws, again using data from period 3. In both panels of Fig 12, empirical quantiles are plotted against theoretical quantiles, the 45° degree line indicating perfect agreement between both sets of quantiles. The Kumaraswamy and beta laws fit the data quite well up to approximately 0.35 and 0.45, respectively. It is then clear that the latter outperforms the former in the sense that it yields better agreement between empirical and theoretical quantiles.

**Table 8:** Goodness-of-fit measures.

Period	Criterion	beta	KW	simplex	UW
1	AIC	−6466.687	−6441.745	−5993.029	−6144.892
	AICc	−6466.681	−6441.739	−5993.023	−6144.887
	BIC	−6455.413	−6430.471	−5981.755	−6133.619
	HQIC	−6462.555	−6437.613	−5988.897	−6140.760
	WIC	−6457.755	−6432.813	−5984.096	−6135.960
	EIC	−6477.157	−6452.015	−6013.894	−6160.247
	AD	2.958	3.170	5.544	3.788
	CVM	0.652	0.657	1.387	0.911
2	AIC	−3287.865	−3266.338	−3076.004	−3033.648
	AICc	−3287.859	−3266.332	−3075.994	−3033.643
	BIC	−3276.584	−3255.058	−3064.720	−3022.368
	HQIC	−3283.731	−3262.204	−3071.866	−3029.515
	WIC	−3278.926	−3257.399	−3067.061	−3024.710
	EIC	−3300.680	−3279.034	−3093.013	−3049.644
	AD	4.031	3.114	5.707	4.181
	CVM	0.674	0.471	0.966	2.120
3	AIC	−4873.815	−4862.928	−4666.304	−4558.565
	AICc	−4873.809	−4862.922	−4666.298	−4558.559
	BIC	−4862.534	−4851.647	−4655.024	−4547.284
	HQIC	−4869.681	−4858.794	−4662.170	−4554.431
	WIC	−4864.876	−4853.989	−4657.365	−4549.626
	EIC	−4885.201	−4875.239	−4680.756	−4571.892
	AD	2.595	2.335	4.443	4.093
	CVM	0.522	0.606	1.020	0.881

## Concluding remarks

The beta distribution is commonly used to model variables that assume values in the standard unit interval. We developed information matrix tests that can be used to test whether the univariate beta model yields an adequate representation of the data. The null hypothesis of correct model specification is tested against the alternative hypothesis that the model specification is in error. The tests seek to verify whether the information matrix equality holds. As is well known, this equality only holds when the model is correctly specified. The tests’ small sample behavior can be improved by using data resampling (bootstrap). We presented the results of extensive Monte Carlo simulations that showed that the tests have good power against different forms of model misspecification, including the case in which the univariate beta model is fitted using data that have an underlying regression structure.

We presented an empirical analysis of Covid-19 mortality rates in the US. We considered three sample periods: (i) before, (ii) before and after, and (iii) after the beginning of the nationwide vaccination drive. The testing inferences indicated that the beta law yields a good representation of the data in the pre-vaccination period. There is also evidence in favor of such a model when mortality rates are computed using data that only cover the vaccination drive period as long as the negative impact of vaccination reach on death rates is moderate; when such an impact is strong, the univariate beta model is rejected. The beta law is also rejected by the information matrix tests when mortality rates are computed using data that cover both periods (before and after the start of the vaccination campaign). The rejection of the beta distribution in this case is due to data heterogeneity.

Our results should be viewed as an initial exploration on the usefulness of information matrix tests for fractional data analysis. The tests we presented proved to be quite useful when applied to the univariate beta model. In future research, we will extend the results presented in this paper to cover other univariate laws that are used to model fractional data (e.g., Kumaraswamy and simplex). We will also seek to extend our results to regression settings, in particular to the beta regression model introduced by [18], and to dynamic beta models, such as the *β*ARMA model introduced by [29, 30]; see also [28, 31]. The beta parameterization used in this paper, which is indexed by mean and precision parameters, will be helpful for the aforementioned extensions of our results.

## Appendix

We present below the quantities required to compute the information matrix test statistics for the beta model. It is possible to show that

$$\begin{array}{cc} & w=\psi^{\prime }\left(\mu \phi \right)+\psi^{\prime }\left(\left(1-\mu \right)\phi \right),\;\;c=\phi \left(\mu w-\psi^{\prime }\left(\left(1-\mu \right)\phi \right)\right),\\ & m=\psi^{\prime \prime }\left(\mu \phi \right)-\psi^{\prime \prime }\left(\left(1-\mu \right)\phi \right),\;\;\frac{\partial \mu^{*}}{\partial \mu }=\phi \psi^{\prime }\left(\mu \phi \right)+\phi \psi^{\prime }\left(\left(1-\mu \right)\phi \right)=\phi w,\\ & \frac{\partial \mu^{*}}{\partial \phi }=\mu \psi^{\prime }\left(\mu \phi \right)-\left(1-\mu \right)\psi^{\prime }\left(\left(1-\mu \right)\phi \right)=\frac{c}{\phi },\\ & \frac{\partial^{2}\mu^{*}}{\partial \phi^{2}}=\mu^{2}\psi^{\prime \prime }\left(\mu \phi \right)-{\left(1-\mu \right)}^{2}\psi^{\prime \prime }\left(\left(1-\mu \right)\phi \right),\\ & \frac{\partial \mu^{†}}{\partial \mu }=-\phi \psi^{\prime }\left(\left(1-\mu \right)\phi \right),\;\;\frac{\partial \mu^{†}}{\partial \phi }=\left(1-\mu \right)\psi^{\prime }\left(\left(1-\mu \right)\phi \right)-\psi^{\prime }\left(\phi \right),\\ & \frac{\partial w}{\partial \mu }=\phi \psi^{\prime \prime }\left(\mu \phi \right)-\phi \psi^{\prime \prime }\left(\left(1-\mu \right)\phi \right)=\phi m,\\ & \frac{\partial w}{\partial \phi }=\mu \psi^{\prime \prime }\left(\mu \phi \right)+\left(1-\mu \right)\psi^{\prime \prime }\left(\left(1-\mu \right)\phi \right),\;\;\frac{\partial c}{\partial \mu }=\phi (w+\phi \frac{\partial w}{\partial \phi }),\\ & \frac{\partial c}{\partial \phi }=\frac{\partial \mu^{*}}{\partial \phi }+\phi \frac{\partial^{2}\mu^{*}}{\partial \phi^{2}}. \end{array}$$

Additionally, we obtain, after some algebra,

$$\begin{array}{c} \nabla D_{n}\left(\theta ;Y\right)=\frac{1}{n}\sum_{t=1}^{n}[\begin{array}{cc} \frac{\partial d_{1}\left(\theta ;Y_{t}\right)}{\partial \mu } & \frac{\partial d_{1}\left(\theta ;Y_{t}\right)}{\partial \phi }\\ \frac{\partial d_{2}\left(\theta ;Y_{t}\right)}{\partial \mu } & \frac{\partial d_{2}\left(\theta ;Y_{t}\right)}{\partial \phi }\\ \frac{\partial d_{3}\left(\theta ;Y_{t}\right)}{\partial \mu } & \frac{\partial d_{3}\left(\theta ;Y_{t}\right)}{\partial \phi } \end{array}], \end{array}$$

where

$$\begin{array}{cc} \frac{\partial d_{1}\left(\theta ;Y_{t}\right)}{\partial \mu } & =\phi^{2}[-\frac{\partial w}{\partial \mu }-2\left(Y_{t}^{*}-\mu^{*}\right)(\frac{\partial \mu^{*}}{\partial \mu })],\\ \frac{\partial d_{1}\left(\theta ;Y_{t}\right)}{\partial \phi } & =2\phi [{\left(Y_{t}^{*}-\mu^{*}\right)}^{2}-w]+\phi^{2}[-2\left(Y_{t}^{*}-\mu^{*}\right)(\frac{\partial \mu^{*}}{\partial \phi })-\frac{\partial w}{\partial \phi }],\\ \frac{\partial d_{2}\left(\theta ;Y_{t}\right)}{\partial \mu } & =-\frac{\partial \mu^{*}}{\partial \mu }-\frac{\partial c}{\partial \mu }-\phi \frac{\partial \mu^{*}}{\partial \mu }[\mu \left(Y_{t}^{*}-\mu^{*}\right)+\left(Y_{t}^{†}-\mu^{†}\right)]\\ & +\phi \left(Y_{t}^{*}-\mu^{*}\right)[\left(Y_{t}^{*}-\mu^{*}\right)-\mu \frac{\partial \mu^{*}}{\partial \mu }-\frac{\partial \mu^{†}}{\partial \mu }],\\ \frac{\partial d_{2}\left(\theta ;Y_{t}\right)}{\partial \phi } & =-\frac{\partial \mu^{*}}{\partial \phi }-\frac{\partial c}{\partial \phi }+\left(Y_{t}^{*}-\mu^{*}\right)[\mu \left(Y_{t}^{*}-\mu^{*}\right)+\left(Y_{t}^{†}-\mu^{†}\right)]\\ & -\phi \frac{\partial \mu^{*}}{\partial \phi }[\mu \left(Y_{t}^{*}-\mu^{*}\right)+\left(Y_{t}^{†}-\mu^{†}\right)]-\phi (Y_{t}^{*}-\mu^{*})(\mu \frac{\partial \mu^{*}}{\partial \phi }+\frac{\partial \mu^{†}}{\partial \phi }),\\ \frac{\partial d_{3}\left(\theta ;Y_{t}\right)}{\partial \mu } & =-\frac{c}{\phi }-\frac{\mu }{\phi }\frac{\partial c}{\partial \mu }+[\psi^{\prime }\left(\left(1-\mu \right)\phi \right)+\left(1-\mu \right)\phi \psi^{\prime \prime }\left(\left(1-\mu \right)\phi \right)],\\ & +2[\mu \left(Y_{t}^{*}-\mu^{*}\right)+\left(Y_{t}^{†}-\mu^{†}\right)][\left(Y_{t}^{*}-\mu^{*}\right)-\mu \frac{\partial \mu^{*}}{\partial \phi }-\frac{\partial \mu^{†}}{\partial \phi }],\\ \frac{\partial d_{3}\left(\theta ;Y_{t}\right)}{\partial \phi } & =\frac{\mu c}{\phi^{2}}-\frac{\mu }{\phi }\frac{\partial c}{\partial \phi }-{\left(1-\mu \right)}^{2}\psi^{\prime \prime }\left(\left(1-\mu \right)\phi \right)+\psi^{\prime \prime }\left(\phi \right)\\ & -2[\mu \left(Y_{t}^{*}-\mu^{*}\right)+\left(Y_{t}^{†}-\mu^{†}\right)](\mu \frac{\partial \mu^{*}}{\partial \phi }+\frac{\partial \mu^{†}}{\partial \phi }). \end{array}$$

Recall that we only use the first two rows of ∇***D***_*n*_(***θ***;***Y***) (evaluated at $\hat{\theta }$) in the information matrix test statistics.
